# Zika virus impairs autophagic flux in trabecular meshwork, and inhibition of autophagy restricts ocular viral transmission and associated pathology

**DOI:** 10.1128/spectrum.01034-25

**Published:** 2025-09-08

**Authors:** Faraz Ahmad, Prince Kumar, Pallav Singh, Trupti Joshi, Pawan Kumar Singh

**Affiliations:** 1 Department of Ophthalmology, Mason Eye Institute, University of Missouri School of Medicine 12271, Columbia, Missouri, USA; 2 MU Institute of Data Science and Informatics, University of Missouri 14716, Columbia, Missouri, USA; 3 Department of Biomedical Informatics, Biostatistics and Medical Epidemiology, University of Missouri 14716, Columbia, Missouri, USA; 4 Christopher S Bond Life Sciences Center, University of Missouri 14716, Columbia, Missouri, USA; University of Florida College of Dentistry, Gainesville, USA

**Keywords:** autophagy, autophagosome, autolysosome, eye, glaucoma, trabecular meshwork, retina, HCQ, Zika virus

## Abstract

Zika virus (ZIKV) is the lone member of Flavivirus family known to cause congenital glaucoma following *in utero* exposure. The molecular mechanisms of ZIKV-induced glaucoma remain elusive, with no known therapeutic modalities. Autophagy plays a dual role in viral infections and glaucoma pathogenesis. However, how ZIKV interferes with autophagic pathways in the trabecular meshwork (TM) and whether autophagy contributes to ZIKV-induced ocular complications remains elusive. Here, we investigated the role of autophagy in ZIKV ocular pathogenesis utilizing a primary human trabecular meshwork cells (HTMC) and IFNAR1^-/-^ mouse model of ocular infection. Our results show that ZIKV permissively infects HTMC, elicits a dysregulated cytokine response, and triggers autophagic activity in TM and the anterior segment of the IFNAR1^-/-^ mouse eyes. Although ZIKV initially activates autophagy in TM, it impairs the overall autophagic flux during infection by modulating the VPS39, a HOPS complex, and STX17, a SNARE complex protein, to inhibit autophagosomal maturation. ZIKV utilizes late endosomes/lysosomes for its replication and egress in TM. We further demonstrated that pharmacologic inhibition of autophagy at the autophagosomal-lysosomal fusion stage by hydroxychloroquine (HCQ), or bafilomycin A1 (Baf-A1), restricts viral replication in TM and ZIKV-induced ocular pathology in mice. Using RNA-seq analysis, we uncovered that autophagy modulation modulates ZIKV-altered host transcriptomes governing critical cellular pathways, including host immunity, antigen processing and presentation, and cell death. Our study is the first to demonstrate the mechanistic insight by which ZIKV manipulates autophagic activity in the ocular milieu, and precise modulation of autophagy could be a potential therapeutic avenue to treat or prevent ZIKV-induced ocular complications.

**IMPORTANCE:**

*In utero* exposure to Zika virus (ZIKV) causes congenital glaucoma; however, the molecular mechanisms of ZIKV-induced glaucoma remain unknown. Here, we demonstrated that ZIKV activates the autophagic activity in the trabecular meshwork and impairs the overall autophagic flux during infection to avoid autolysosomal maturation and uses late-endosomes/lysosomes for their egress. Inhibition of autophagy at late stages, specifically at the autophagosomal-lysosomal fusion stage, restricts viral replication and ZIKV-induced ocular complications.

## INTRODUCTION

Zika virus (ZIKV) was first discovered in 1947 in Zika forests of Uganda; it gained global attention in 2015 through an epidemic in the Americas. It is currently circulating in 89 countries and is a global public health threat with the potential to cause pandemics (1). ZIKV is an arbovirus that causes severe congenital ocular abnormalities along with other congenital birth defects such as microcephaly, intrauterine growth restriction, placental insufficiency, and fetal demise (collectively known as congenital Zika syndrome, CZS) in fetuses and infants from *in utero* exposure and Guillain-Barré syndrome in adults (2). The involvement of the eye in ZIKV infection was first reported during the 2015 epidemic, where infants born with microcephaly showed evidence of severe congenital ocular abnormalities, including chorioretinal atrophy, pigment mottling, macular scarring, intraocular calcifications, microphthalmia, anophthalmia, iris coloboma, lens subluxation, cataract, optic nerve damage (increased cup: disk ratio, optic nerve pallor, optic nerve hypoplasia), and congenital glaucoma (3–8). In addition to clinical studies, several experimental studies from our laboratory and others have demonstrated ZIKV tropism toward different ocular tissues and pathological manifestations in various animal models (2, 9–13). However, the molecular mechanism of ZIKV-induced ocular manifestations in general and congenital glaucoma in particular, remains unknown. In addition, currently, no vaccines or therapeutics are available to avert or treat ZIKV congenital ocular infection; therefore, insight into the cellular and molecular mechanisms of ZIKV ocular infection would contribute to the development of effective treatment for affected individuals.

Autophagy has been shown to play a crucial role in virus-host interaction and is expected to check viral infection at multiple levels by inhibiting viral replication, regulating inflammatory immune and antiviral IFN response, and promoting antigen presentation (14, 15). However, many RNA viruses have evolved to manipulate autophagy for immune evasion, replication, assembly, and release from infected cells (14, 15). Autophagy is an evolutionarily conserved eukaryotic cellular process involving the formation of double-membrane vesicles called autophagosomes, which carry damaged or infected cellular cargo (16, 17). These autophagosomes fuse with endosomes to form amphisomes (an intermediate autophagic compartment), followed by fusion with lysosomes to form autolysosomes, which eventually degrade the incoming cellular waste cargo. The overall balance between the formation of autophagosomes and the degradation of autolysosomes dictates the fate of autophagic flux (16). Multiple viruses, for example, ZIKV, Dengue virus (DENV), Herpes Simplex virus, Japanese encephalitis virus, and Hepatitis C virus, have been shown to hijack/impair the overall process of autophagic flux for their replication and survival (18–23). Recent studies have shown that ZIKV induces autophagy in placental cells and fetal neural stem cells to mediate viral replication (19, 21). In the eye, the trabecular meshwork (TM) regulates the aqueous humor outflow and maintains the intraocular pressure (IOP). Pathological stressors can lead to TM dysfunction, resulting in IOP elevation and glaucoma. We recently demonstrated that ZIKV replicates in the TM and induces trabeculitis in mice (10). However, whether the ZIKV exploits autophagic machinery to promote viral replication and associated pathology in TM and the ocular milieu remains unknown.

In this study, we investigated the role of autophagy in ZIKV infection in TM and its impact on ZIKV-induced ocular complications. We demonstrated that ZIKV infection initiates autophagic activities in the primary human trabecular meshwork cells (HTMC) and anterior segments of the mouse eyes. Inhibition of autophagy in HTMC at the autolysosomal maturation stage attenuates viral replication. We also demonstrated that ZIKV impairs the autophagic flux to avoid autolysosomal maturation and uses late endosomes/lysosomes as replication hubs. Consistent with these findings, we showed that activation of autophagy by rapamycin enhances the ZIKV-induced ocular complications, whereas inhibition by hydroxychloroquine (HCQ) restricts the ZIKV-induced ocular damage in IFNAR1^-/-^ mice. Collectively, our findings, for the first time, demonstrate a mechanistic insight into the role of autophagy in ZIKV TM infectivity and associated ocular pathogenesis and provide a foundation for developing therapeutics to treat or control ZIKV-induced ocular manifestations.

## RESULTS

### ZIKV permissively infects TM cells and evokes a dysregulated inflammatory cytokine response

**Fig 1:**
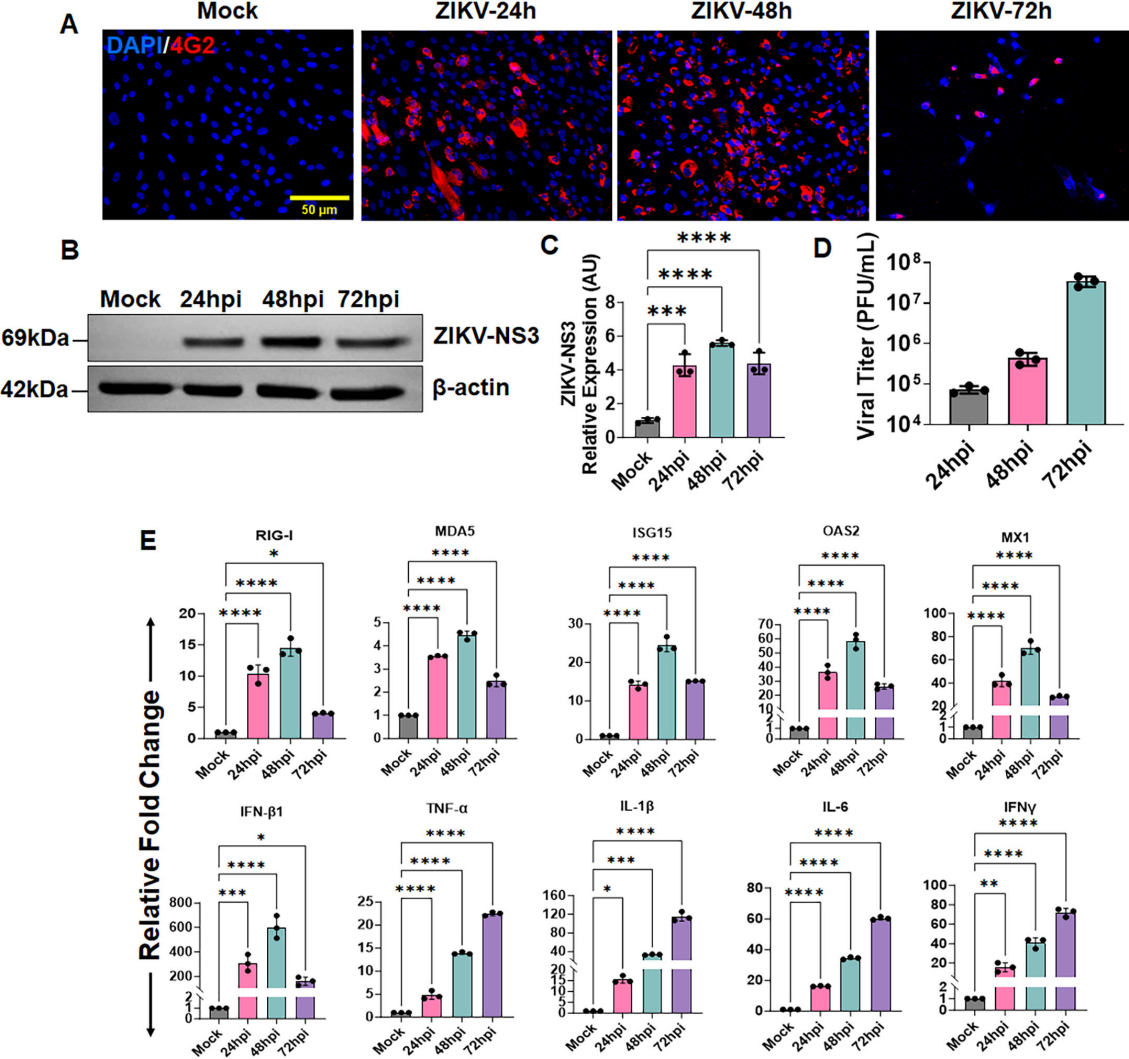
ZIKV permissively infects HTMCs and elicits a dysregulated immune response. (**A**) Primary HTMCs were infected with ZIKV strain PRVABC59 at a multiplicity of infection (MOI) of 1 for the indicated time points. Mock-treated cells were used as controls. Infected and mock-treated cells were fixed and immunostained for anti-flavivirus E antigen antibody 4G2. The representative images show the presence of ZIKV (Red) and DAPI-stained nucleus (Blue), scale bar, 50 µm. (**B**) In a second set of experiments, ZIKV-infected and mock-treated cells were subjected to immunoblotting for ZIKV NS3 protein. (**C**) Densitometric analysis of NS3 immunoblots was performed using ImageJ. The bar graph represents the mean ± SD of three biological replicates. (**D**) The culture supernatants from infected cells were subjected to plaque assay, and the plaque counts were presented as PFU/mL. (**E**) In another set of experiments, ZIKV-infected and mock-treated HTMC were subjected to qPCR for the indicated gene expression. The bar graph represents means ± SD from three biological replicates. *P  <  0.05; **P  <  0.005; ***P  <  0.0005; ****P  <  0.0001 (one-way ANOVA; mock vs ZIKV).

*In utero* exposure to ZIKV is known to cause elevated IOP and congenital glaucoma in neonates and infants (6, 7, 16). In the eye, TM regulates IOP by controlling the aqueous humor outflow, and TM dysfunction results in IOP elevation. Recently, we demonstrated that ZIKV induces trabeculitis and glaucomatous pathology, including IOP elevation and retinal ganglion cell (RGC) loss in murine models (10). In this study, to investigate the molecular mechanisms behind ZIKV-induced glaucomatous pathology, we first tested the ZIKV infectivity toward TM and its impact on host immune/anti-viral response. For this study, we used the ZIKV Asian lineage strain PRVABC59. Although ZIKV African strains are more virulent, Asian strains are responsible for recent epidemics in the Americas. Asian strains cause slow infection with a lack of early cell death, which contributes to their ability to cause persistent central nervous system infection of fetuses, leading to congenital microcephaly (24). We challenged human primary TM cells (HTMC) with the ZIKV epidemic strain PRVABC59 in a time-course study. Our results show that ZIKV productively replicates in HTMC, as evidenced by an increased flaviviral envelope (E) antigen: 4G2, positivity by IFA (Fig. 1A; Fig. S1A). We further confirmed the viral infectivity in TM cells by immunoblotting using ZIKV NS3 protein, and our results show a time-dependent NS3 positivity in these cells (Fig. 1B and C). We observed a viral peak at 48 hours post-infection (hpi), which decreased by 72 hpi with increased cytopathy (Fig. S1B). To confirm ZIKV replication in HTMC and production of infectious virions, we quantified ZIKV progeny from the culture supernatant in a time-course study via plaque assay. Our results show a time-dependent increase in ZIKV plaques, indicating permissive replication of ZIKV in HTMC (Fig. 1D). Together, these results confirmed that ZIKV can efficiently invade and replicate in TM cells.

Viral pathogens are known to modulate host immunity by regulating pattern recognition receptors (PRRs) and several inflammatory and anti-viral mediators that generally act to curb the pathogen spread. However, a dysregulated immune response may contribute to exacerbated pathology. Considering this, we next sought to investigate whether ZIKV infection induces inflammatory and anti-viral responses in TM cells. To this end, our qPCR analysis showed that ZIKV infection induces the expression of PRRs (RIG-I, MDA5) in HTMC, which are known to sense RNA viruses (Fig. 1E). The level of these PRRs exhibited a rise over mock with a peak expression at 48 hpi, which slightly declined by 72 h time interval. Activation of PRRs results in the production of downstream effector molecules. Here, we observed that the upregulation of these PRRs resulted in the induction of prominent anti-viral mediators (ISG15, OAS2, and MX1) and signature type 1 interferon, IFN-β1, with a peak expression at 48 hpi. Interestingly, the expression of acute phase inflammatory cytokines (TNF-α, IL-1β, and IL-6) and type II interferon, IFNγ, showed a time-dependent increase in HTMC following ZIKV infection (Fig. 1E). The peaking of these acute pro-inflammatory cytokines concomitant with decreased expression of dominant anti-viral effectors (ISG15, OAS2, MX1, and IFN-β1) at late time point (72 hpi) indicates an uncontrolled viral spread and a dysregulated immune response in TM.

### ZIKV activates autophagy in the human trabecular meshwork

**Fig 2:**
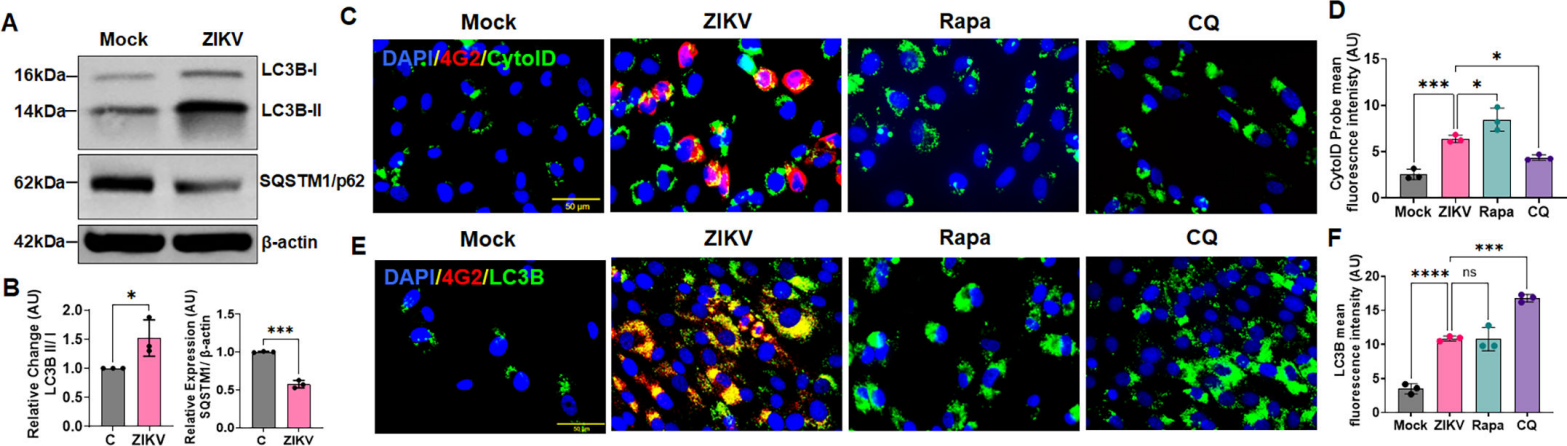
ZIKV activates autophagic activity in HTMCs. HTMCs were infected with ZIKV at MOI of 1 for 48 h. Mock-treated cells were used as controls. (**A**) Infected and mock-treated cells were subjected to immunoblotting for autophagy markers LC3B and SQSTM1/p62. (**B**) Densitometric analysis for LC3B-II/I and SQSTM1/p62 immunoblots was performed using ImageJ. The bar graph represents the mean ± SD of three biological replicates. ***P  <  0.0005; ****, *P*  <  0.0001 (unpaired t-test; C vs ZIKV). (**C**) Autophagic activation was further evaluated in ZIKV-infected/mock-treated HTMC by staining with a CYTO-ID autophagy detection probe, which is specific for autophagosomes (Green labeling). An autophagy activator, rapamycin (Rapa), and an autophagy inhibitor, chloroquine (CQ), without ZIKV infection, were used as autophagy inducer and inhibitor controls, respectively, in these experiments. (**D**) The green fluorescence for CYTO-ID probes was quantified using ImageJ and presented as mean fluorescence intensity. (**E**) In another set of experiments, ZIKV-infected and mock-treated cells were immunostained with autophagy marker LC3B (Green) along with anti-flavivirus E antigen antibody 4G2 (Red), and DAPI nuclear stain (Blue). Rapa and CQ, without ZIKV infection, were used as autophagy inducer and inhibitor controls, respectively. (**F**) The LC3B punctate fluorescence was measured using ImageJ and presented as mean fluorescence intensity. Scale bar, 50 µm.

Autophagy is an evolutionarily conserved eukaryotic host cell response against many pathogens, including RNA viruses (16, 25, 26). Both bacterial and viral pathogens are vulnerable to autophagic destruction; however, many successful pathogens, particularly RNA viruses, evolve strategies to circumvent autophagy and use it for their replication and spread (19, 21, 25, 26). Autophagy plays a critical role in innate immunity; moreover, it can be modulated by factors that can skew the host immune response, such as cytokines. We observed that ZIKV evokes a dysregulated inflammatory cytokine response in TM; therefore, we postulated that ZIKV may exploit autophagic pathways in TM cells to facilitate its replication in the eye. To confirm this, we first infected HTMC cells with ZIKV and assessed the autophagy activation by immunoblotting using autophagy markers, microtubule-associated-protein light-chain 3 (LC3B) and Sequestosome 1 (SQSTM1)/p62. LC3B converts from soluble LC3B-I to the lipidated form LC3B-II upon autophagy activation, which is essential for autophagosome formation, whereas degradation of SQSTM1/p62 indicates autophagy activation (17, 27). We observed that ZIKV infection in human primary TM cells resulted in a marked increase in the expression levels of lipidated LC3B-II, along with the degradation of SQSTM1/p62, suggesting activation of autophagy and increased autophagosome biogenesis (Fig. 2A and B). To further confirm the ZIKV-induced autophagic activation in HTMC, we used a CYTO-ID autophagy detection kit (Enzo Life Science) that uses a cationic amphiphilic tracer (CAT) probe that rapidly partitions into cells with induced phospholipidosis and selectively labels the accumulated autophagic vesicles/autophagosomes. Our results confirmed that ZIKV induces autophagic activation in TM, as evident by increased levels of autophagosome staining (Green fluorescence) by CYTO-ID dye in ZIKV-infected cells (Fig. 2C and D; Fig. S2A). The punctate staining pattern in ZIKV-infected cells was characteristically comparable to cells treated with rapamycin, a positive regulator of autophagy, as opposed to a bright floral fluorescence exhibited in cells treated with an autophagy inhibitor, chloroquine (Fig. 2C). Moreover, we measured the LC3B accumulation along with ZIKV-E antigen (4G2) by employing immunofluorescence staining, and our results further confirmed the activation of autophagy in HTMCs with ZIKV infection (Fig. 2E and F; Fig. S2B). In these assays, we used known autophagy modulators, rapamycin (Rapa, an inducer) and chloroquine (CQ, an inhibitor), which, respectively, show increased accumulation and decreased degradation of LC3B in autophagosomes. Collectively, these results suggest that ZIKV activates autophagy and autophagosome formation in the human trabecular meshwork.

### ZIKV infection impaired autophagic flux in the human trabecular meshwork

Once autophagy is activated, the autophagosome is formed by assembly of cellular membranes known as phagophores, followed by sequestration and engulfment of damaged or infected organelles targeted for autophagic recycling in these double-membrane vesicles. The autophagosomes fuse with endosomal vesicles and vacuolar ATPases that acidify these vesicles, called amphisomes. These acidified amphisomes/autophagosomes then fuse with lysosomes to form mature autolysosomes, also known as autolysosome maturation. Finally, autolysosomes degrade and recycle cellular cargo, which completes the process of autophagic flux. Most viruses that benefit from autophagic induction prevent autophagic flux and block autolysosomal maturation for their survival and spread (16, 28).

**Fig 3:**
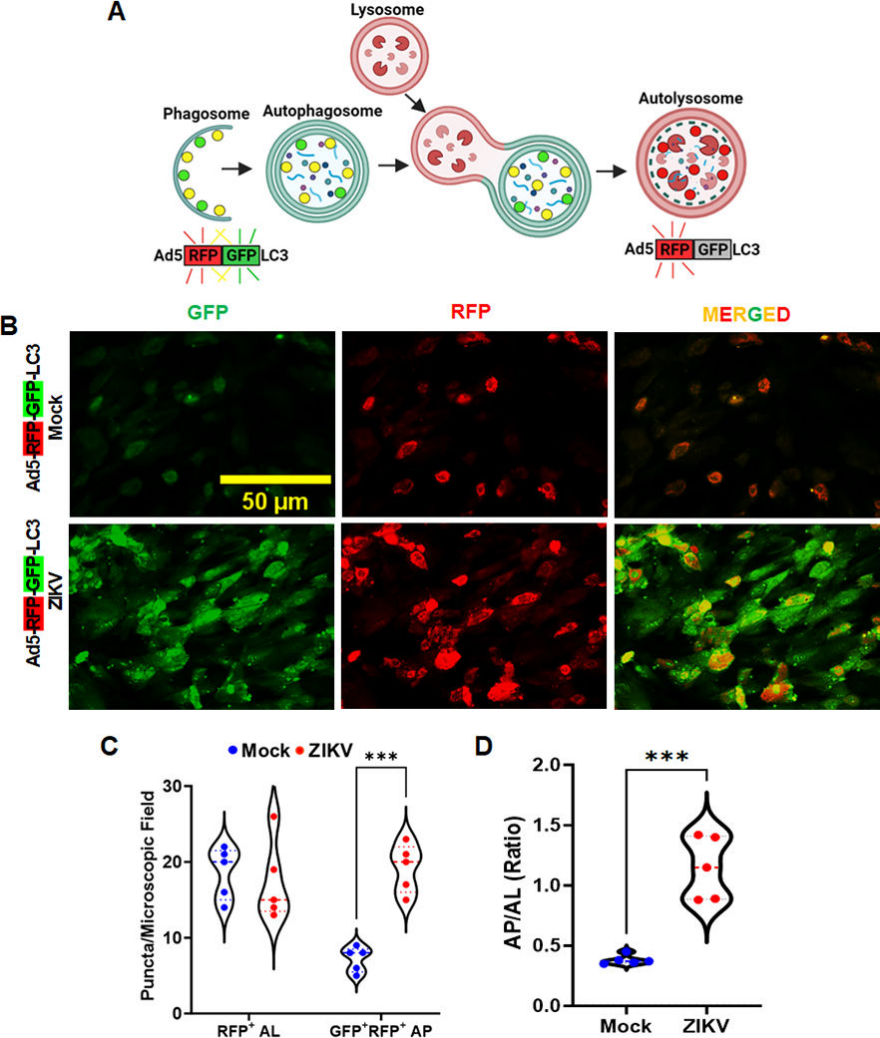
ZIKV infection impaired autophagic flux in HTMCs. (**A**) Schematic showing Ad5-mRFP-GFP-LC3 construct fluorescing yellow-green (eGFP/mRFP) at the neutral pH in autophagosomes vs. red (mRFP) at acidic pH in autolysosomes. eGFP fluorescence is quenched in autolysosomes at an acidic pH (pH <6.0). (**B**) HTMCs were transduced with Ad5-mRFP-GFP-LC3 construct for 24 hours and subsequently infected with ZIKV at a multiplicity of infection (MOI) of 1 for another 24 hours. Ad5-mRFP-GFP-LC3 vector alone (without ZIKV infection) transduced cells were used as a mock control. The slides were briefly fixed (5 min) with paraformaldehyde and imaged using a Keyence fluorescence microscope. The yellow-green puncta indicate RFP^+^GFP^+^LC3 (autophagosomes), and the red puncta indicate RFP^+^LC3 (autolysosome), scale bar, 50 µm. (**C**) The violin plot denotes the quantification of RFP^+^ autolysosomes (AL) and RFP^+^GFP^+^ autophagosomes (AP) in mock and ZIKV-infected cells. (**D**) The violin plot represents the AP/AL ratio in Ad5-mRFP-GFP-LC3-transduced cells with (ZIKV) or without (mock) treatment. The data are representative of 10–15 fields/groups from two independent experiments. The schematic in panel A was created using BioRender software (BioRender.com).

Since we observed that ZIKV activates autophagy in TM, next, we sought to investigate whether ZIKV blocks autophagic flux to evade autolysosomal degradation. To test this, we utilized a pH-sensitive mRFP-GFP-LC3 expressing Adenoviral vector (Ad5-mRFP-GFP-LC3) that upon cellular transduction, labels LC3 as yellow puncta (positive for both GFP and RFP) in autophagosomes or un-acidified amphisomes and RFP-only in acidified amphisomes or autolysosomes (Fig. 3A). Measurement of red/yellow puncta (autolysosomes [AL]/autophagosomes [AP]) per cell determines the rate of autophagic flux. To determine the autophagic flux in TM, we transduced human primary TM cells with Ad5-mRFP-GFP-LC3 for 24 hours and subsequently exposed them to ZIKV for another 24 hours. Ad5-mRFP-GFP-LC3-transduced cells without ZIKV infection served as mock control. We found that ZIKV infection in HTMCs markedly increased the yellow/green (RFP^+^GFP^+^/GFP^+^-LC3) puncta (Fig. 3B), indicating an increased autophagosome accumulation. By contrast, control cells exhibited only red fluorescence (RFP^+^-LC3), representing autolysosomes. The total number of RFP^+^GFP^+^-LC3 puncta and overall AP/AL ratio was also significantly increased in ZIKV-infected cells in contrast to controls (Fig. 3C and D). These findings indicate that ZIKV reduces or impairs autophagic flux in TM cells during infection.

### ZIKV blocks autophagic maturation and uses autophagosomes/late endosomes/lysosomes for its replication and egress in TM

**Fig 4:**
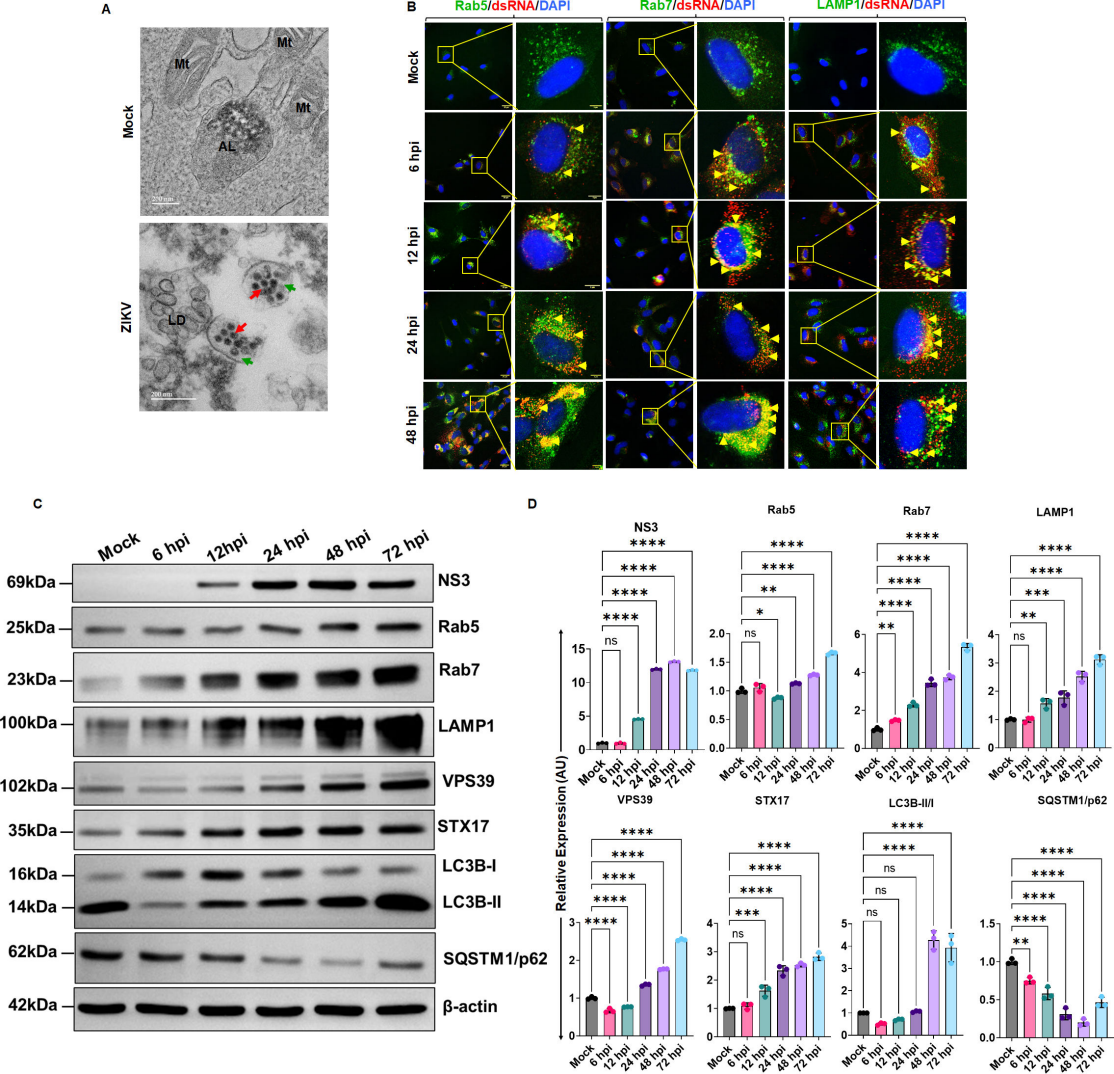
ZIKV blocks autophagic maturation and replicates in autophagosomes, late endosomes, and lysosomes. HTMCs were infected with ZIKV strain PRVABC59 at a multiplicity of infection (MOI) of 1 for the indicated time points. The cells were fixed and subjected to (**A**) Transmission electron microscopy analysis to assess ZIKV localization in autophagosomes. Representative micrographs show ZIKV (Red arrow) localized in autophagosomes (Green arrow). Mt: mitochondria, AL: Autolysosomes, LD: lipid droplets. Scale bar 200 nm. (**B**) From another set of experiments, HTMCs were coimmunostained with viral anti-dsRNA antibody (Red) with cellular organelle markers Rab5 (early endosome), Rab7 (late endosome), and LAMP-1 (lysosome) (Green), and DAPI nuclear stain (Blue). The representative confocal images show co-localizations of dsRNA with organelles at various time points (a few representatives indicated with yellow arrows). Scale bar, 25 µm (left panels), 5 µm (right panels). (**C**) In another set of time-dependent experiments (6, 12, 24, 48, and 72 h), ZIKV-infected and mock-treated cells were subjected to immunoblotting for ZIKV NS3 protein, organelle markers (Rab5/Rab7/LAMP1), HOPS complex protein (VPS39), a SNARE complex protein (STX17), along with autophagy marker proteins LCB and SQSTM1/p62. (**D**) Densitometric analysis of NS3, Rab5, Rab7, LAMP1, VPS39, STX17, LC3B-II/I, and SQSTM1/p62 immunoblots was performed using ImageJ. The bar graph represents the mean ± SD of three biological replicates. *P  <  0.05; **P  <  0.005; ***P  <  0.0005; ****P  <  0.0001 (two-way ANOVA).

Manipulation of autophagosome fusion and maturation events is an essential regulatory step often exploited by RNA viruses for their survival. Multiple viruses, including flaviviruses such as ZIKV (20, 21), DENV (29), and β-coronaviruses, for example MHV (30), SARS-CoV-2 (31–33), reportedly inhibit autophagic maturation/autolysosome formation and impair lysosomes/late endosomes equipped to kill most pathogens, and instead utilize these compartments for their replication and egress. ZIKV NS3-NS2B protease interaction has been shown to inhibit endosome-lysosome fusion (34). The observation that ZIKV activates autophagy and blocks autophagic flux to avoid autolysosomal degradation in TM cells leads us to hypothesize that ZIKV may use autophagic intermediate vesicles for its replication and egress by preventing late autophagic vacuole maturation. To investigate this, first, we performed transmission electron microscopy (TEM) analysis to visualize the ZIKV localization within autophagosomes. Our TEM results show that ZIKV is localized in autophagosomes (Fig. 4A). To further confirm whether ZIKV replicates in the autophagic intermediate organelles and not just uses them for trafficking, we performed a time-course (6, 12, 24, 48, and 72 h) infection study. Following infection, we coimmunostained the infected and mock-treated cells with viral dsRNA-specific antibody with organelle markers Rab5 (early endosomes), Rab7 (late endosomes), and LAMP1 (lysosomes). dsRNA is a replication intermediate formed during ZIKV/ RNA viral replication (35). Our results show that ZIKV indeed replicates in these autophagic intermediate organelles (endosomes/lysosomes) as revealed by the dsRNA co-localization with Rab5^+^/Rab7^+^/LAMP1^+^ organelles (Fig. 4B; Fig. S4B). To further visualize the intracellular viral presence in these organelles, we also performed coimmunostaining for ZIKV-E antigen (4G2) with Rab5/Rab7/LAMP1. Our results confirm the presence of ZIKV in all these compartments, as indicated by a time-dependent increase in ZIKV-4G2 positivity with these organelles (Fig. S3 and S4A), confirming our notion that ZIKV uses autophagic intermediate compartments for its replication and egress. The ability of ZIKV to replicate in endo-lysosomal compartments motivated us to investigate the virus’s ability to impair auto-lysosomal maturation, which ultimately inhibits autophagic flux. To further confirm this, we performed immunoblotting in a time-course infection study for various organelle-specific markers, Rab5, Rab7, and LAMP1, alongside markers implicated in autolysosomal maturation, including STX17 and VPS39. Our results show that ZIKV significantly induced the expression of Rab5, Rab7, and LAMP1 in a time-dependent fashion concomitant with infection progression (Fig. 4C and D). The viral infection dynamics in different autophagic compartments and induced expression of Rab5/Rab7/LAMP1 markers suggest that ZIKV induces endosome/lysosome biogenesis and can utilize these compartments for its egress. Furthermore, VPS39 (a HOPS-specific subunit that interacts with Rab7) and STX17 (a SNARE complex protein that translocates to autophagosomes when they are close and disappears following autolysosomal maturation) (35) also showed a time-dependent activation upon ZIKV infection, indicating increased autophagosome biogenesis (Fig. 4C and D). Together, activation of these organelle markers along with consistent induction of HOPS (VPS39) and SNARE (STX17) complex proteins indicates increased biogenesis of autophagic vacuoles and inhibition of autolysosomal maturation upon viral infection in TM cells.

To further test whether the increased biogenesis of autophagosomes/late-autophagic compartments resulted in an increased autophagic turnover and blockade of autophagosome maturation into autolysosomes, we tested the accumulation of LC3B-II and SQSTM1/p62 in these time-dependent infection studies. Our results showed a time-dependent accumulation of lipidated LC3B-II in ZIKV-infected HTMC (Fig. 4C and D). The SQSTM1/p62 protein showed a time-dependent degradation up to 48 h and tended to initiate accumulation by 72 h (Fig. 4C and D) (we were unable to track beyond 72 h due to severe ZIKV-induced cytopathy, Fig. S1B), suggesting impaired autophagic activity during infection progression. Altogether, these findings confirm that ZIKV impaired autophagic flux and blocked autolysosomal maturation with an increased autophagosome accumulation, which served as a viral replication niche in TM cells.

### Autophagy induction promotes ZIKV replication, and suppression of autolysosomal maturation restricts viral infection in TM

**Fig 5:**
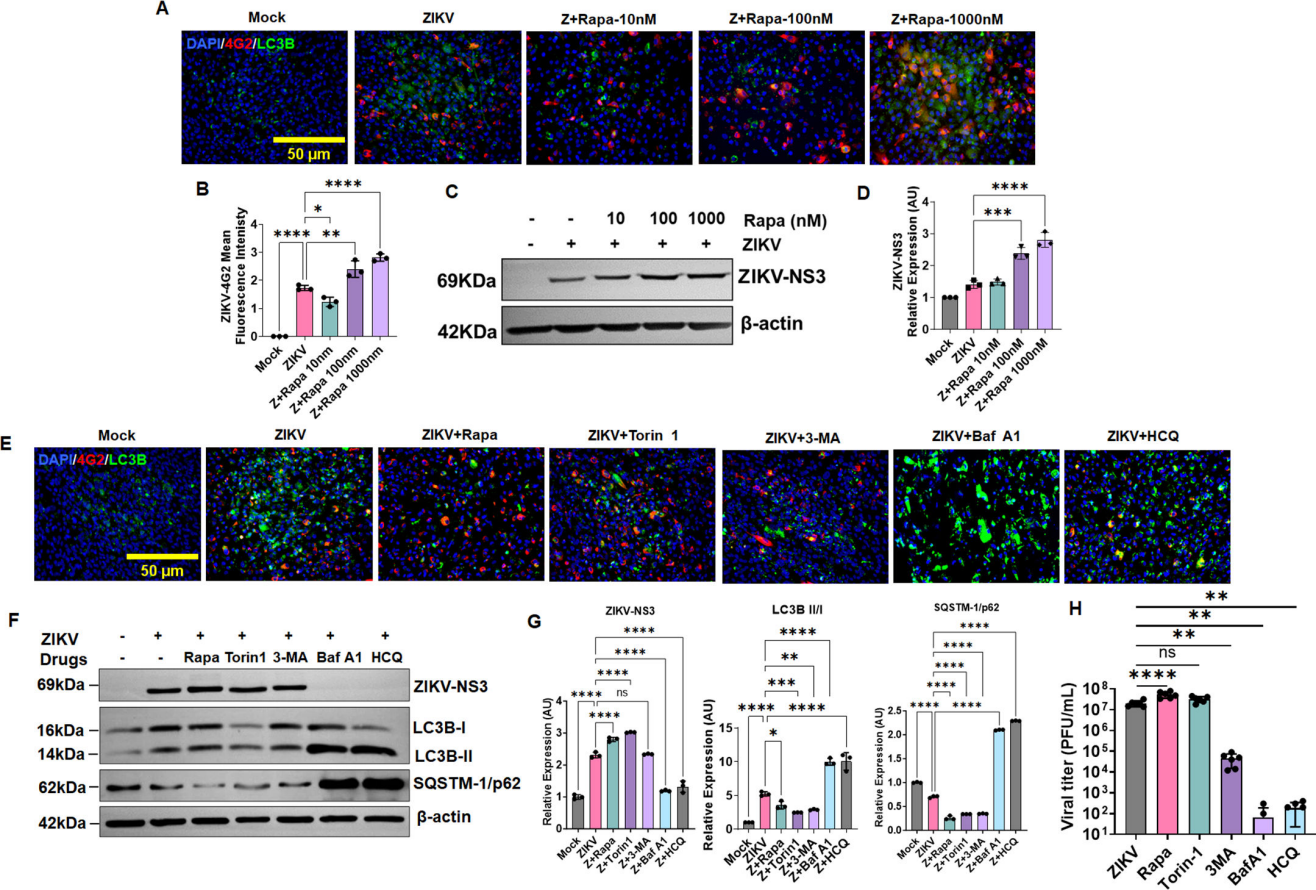
Autophagy induction promotes ZIKV replication, while autophagy suppression restricts viral replication in HTMCs. (**A**) HTMCs were pretreated with different doses (10, 100, and 1,000 nM) of autophagy inducer rapamycin (Rapa) followed by ZIKV (Z) infection (multiplicity of infection [MOI]:1) for 48 h. Mock-treated cells were used as controls. Cells were fixed and immunostained for anti-flavivirus E antigen antibody 4G2, autophagy marker LC3B (green), and DAPI nuclear stain (Blue), scale bar, 50 µm. (**B**) The 4G2 fluorescence intensity was measured using ImageJ and presented as mean fluorescence intensity. (**C**) In another set of experiments, ZIKV and Rapa-treated cells were subjected to immunoblotting for ZIKV NS3 proteins. (**D**) Densitometric analysis was performed using ImageJ, and the bar graph represents ZIKV (Z) NS3 protein quantification, normalized to β-actin from three biological replicates (mean ± SD, ***P  <  0.0005; ****P  <  0.0001, one-way ANOVA; C vs. treatment). (**E**) HTMCs were pretreated with various autophagy activators (Rapa [1 µM], Torin 1 [1µM]) and inhibitors (3-MA [2 µM], Baf-A1 [50 nM], and HCQ [50 µM]), followed by ZIKV infection (MOI: 1) for 48 h. The cells were fixed and immunostained for anti-flavivirus E antigen antibody 4G2 (Red), autophagy marker LC3B (green), and DAPI nuclear stain (Blue), scale bar, 50 µm. (**F**) In another set of similar experiments, protein lysates from ZIKV-infected cells in the presence and absence of respective drugs were subjected to immunoblotting for ZIKV (Z) NS3 protein and autophagy markers LC3B-II and SQSTM1/p62. (**G**) Densitometric analysis for ZIKV (Z) NS3, LC3B-II/I, and SQSTM1/p62 proteins (relative to β-actin) (*n* = 3) were performed using ImageJ (mean ± SD,*P  <  0.05; **P  <  0.005; ***P  <  0.0005; ****P  <  0.0001; one-way ANOVA; C vs. ZIKV and ZIKV vs. treatment). (**H**) The culture supernatants from ZIKV-infected and rapamycin, torin-1, 3-MA, Baf-A1, and HCQ-treated cells (48 hpi) were subjected to plaque assay. The plaques were counted and expressed as PFU/mL (*n* = 3 in duplicate, data represent mean ± SD, **P  <  0.005; ****P  <  0.0001; ns: no significant, one-way ANOVA; ZIKV vs. treatment).

Autophagy modulation has been shown to play a vital role in the viral replication and pathogenesis of several RNA viruses (25, 26). After an overall detection of ZIKV-induced signaling cascades related to autophagic induction in TM, we sought to determine whether autophagy manipulation contributes to viral replication in TM. To investigate this, we pharmacologically modulated autophagy in TM with widely used autophagy activators: Rapamycin (Rapa), Torin-1, and autophagy inhibitors: 3-Methyladenine (3-MA), Bafilomycin-A1 (Baf-A1), and Hydroxychloroquine (HCQ). First, we titrated the doses of these compounds for their toxicity profile and used a safe and effective dosage for the rest of the assay (Fig. S5A). The autophagy modulation by these drugs was also confirmed without ZIKV infection by immunoblotting for LC3B lipidation and SQSTM1/p62 degradation (Fig. S5B and C). For autophagy activation studies, first, we used different doses of rapamycin (10, 100, and 1,000 nM) to pretreat TM cells before ZIKV infection and assessed the ZIKV replication by IFA and western blotting. Our immunofluorescence staining analysis revealed a dose-dependent increase in ZIKV replication as evidenced by increased 4G2 positivity in HTMCs (Fig. 5A and B; Fig. S6A). The western blot analysis further confirmed a dose-dependent increase in ZIKV NS3 protein in the presence of rapamycin (Fig. 5C and D), corroborating our hypothesis that autophagy activation supports ZIKV replication in TM cells.

Next, we inhibited autophagy using stage-specific autophagy inhibitors and measured the viral infectivity in TM cells. We infected HTMCs in the presence and absence of 3-MA, Baf-A1, and HCQ. 3-MA inhibits autophagy at a very early stage by blocking autophagosome formation through the inhibition of type III Phosphatidylinositol 3-kinase (PI-3K), whereas Baf-A1 and HCQ block autophagy at late stages by blocking the fusion of autophagosomes with lysosomes (19, 36). In these experiments, once again, we used rapamycin and Torin 1 as autophagy inducer controls (both drugs modulate autophagy at the initiation stage and promote autophagosome formation). Our immunofluorescence (Fig. 5E; Fig. S6B) and western blot (Fig. 5F and G) analysis revealed a remarkable reduction in ZIKV replication by Baf-A1 and HCQ treatment; in contrast, 3-MA treatment exhibited only a marginal decrease in viral replication. The immunofluorescence staining in infected TM confirmed reduced ZIKV-E antigen, 4G2, with increased LC3B in cells treated with autophagy inhibitors and increased 4G2-positive cells with activators (Fig. 5E). Similarly, western blot data displayed reduced ZIKV-NS3 expression with autophagy inhibitors and increased expression with activators (Fig. 5F and G). The activation and inhibition of autophagy by respective activators and inhibitors in the presence and absence of ZIKV were confirmed by LC3B-II and SQSTM1/p62 accumulation/degradation by western blotting (Fig. 5F and G; Fig. S5B and C). To further assess the effect of autophagy modulation on ZIKV progeny production, we performed the plaque assay from HTMC culture supernatant that was infected in the presence and absence of autophagy modulators, rapa, torin-1, 3-MA, Baf-A1, and HCQ. Consistent with our immunoblotting and immunofluorescence analysis, the plaque assay results confirm that Baf-A1 and HCQ treatment significantly inhibited the ZIKV plaque formation (Fig. 5H). In contrast, rapamycin treatment significantly increased the number of infectious virus particles in comparison to ZIKV-infected (untreated) cells (Fig. 5H). The number of ZIKV plaques in Torin-1-treated groups was comparable to ZIKV, while 3-MA treatment showed a significant reduction in plaque counts in comparison to ZIKV-infected cells. Collectively, these findings suggest that ZIKV mainly uses late-stage autophagic compartments for its replication/spread, and autophagy inhibition can restrict ZIKV infection.

### ZIKV infection induces autophagic activity in the ocular milieu, and autophagy inhibition ameliorates ZIKV-induced ocular complications

**Fig 6:**
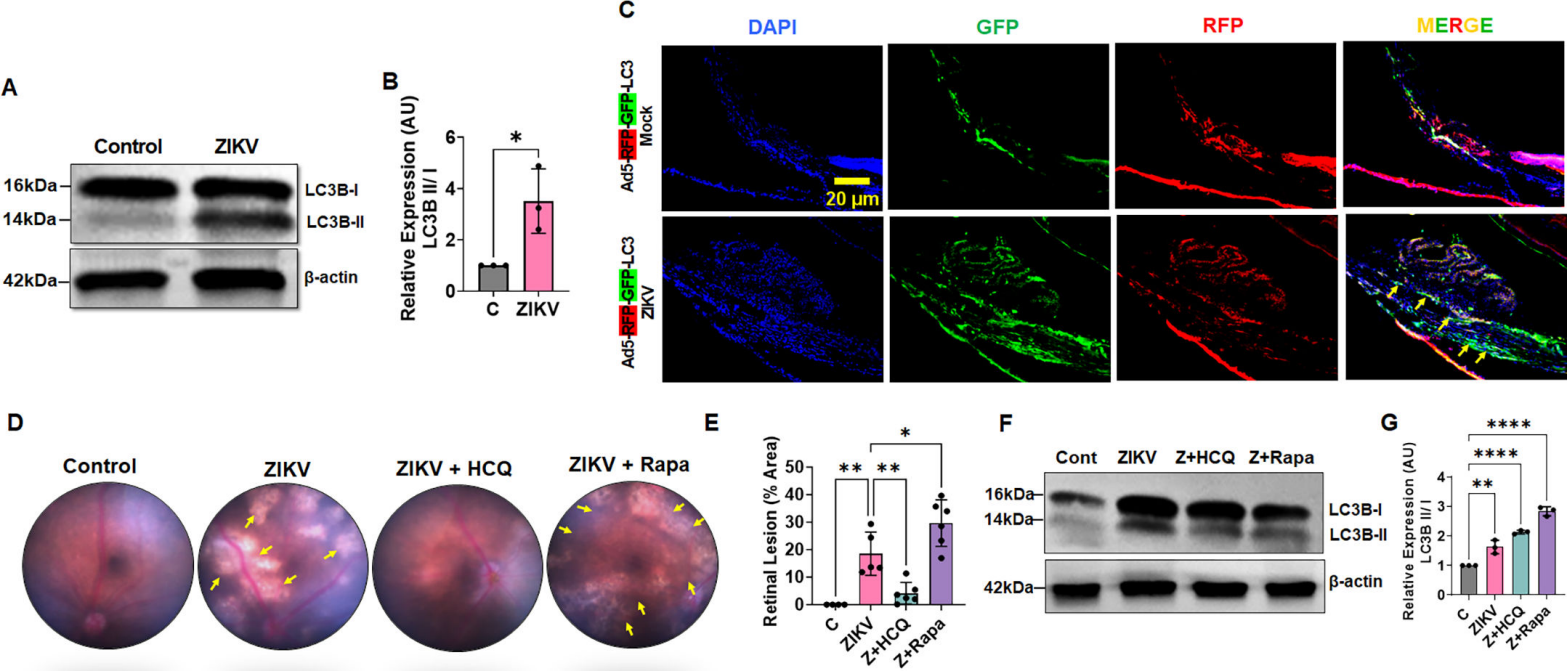
ZIKV impairs autophagic flux in the mouse anterior segment, and lysosomal inhibitors of autophagy ameliorate ZIKV-induced ocular pathology. (**A**) Eyes of 6- to 8-week-old IFNAR1^−/−^ mice (*n* = 6-12/group) were infected with ZIKV PRVABC59 (1 × 10^4^ PFU/eye) by anterior chamber (AC) injections. Saline-injected eyes were used as a control. Seven days post-infection, AS tissue lysates were subjected to immunoblotting for autophagy marker LC3B. (**B**) Densitometric analysis of LCB-II/I ratio (normalized to β-actin) was performed using ImageJ. Each data point represents mean ± SD from two pooled eyes from three biological replicates (*n* = 6 eyes/group). ***P  <  0.0005; t-test; C vs. ZIKV. (**C**) IFNAR1^−/−^ mice eyes were transduced with Ad5-mRFP-GFP-LC3 via intravitreal injections. Three days post-Ad5 transduction, the eyes of the mice were infected with ZIKV (1 × 10^4^ PFU/eye) by AC injection. Seven days post-ZIKV infection, eyes were enucleated, and 10 µM thin cryosections were imaged for GFP^+^ or GFP^+^/RFP^+^LC3 (autophagosomes pointed with yellow arrows) with DAPI nuclear stain (Blue) in the anterior segment of the eye, scale bar, 20 µm. (**D**) IFNAR1^−/−^ mice were pretreated with either HCQ (40 mg/kg/day) or rapamycin (1 mg/kg/day) via intraperitoneal route 1 day before ZIKV infection, followed by three consecutive days of treatment post-ZIKV infection. Both treated and untreated mice eyes were infected with ZIKV (1 × 10^4^ PFU/eye) by AC inoculation. Seven days post-ZIKV infection, the retina was imaged using Micron IV. Representative funduscopic images showing retinal lesions/chorioretinal atrophy (pointed with yellow arrows) in treated/untreated groups. (**E**) The percentage of areas with retinal lesions in ZIKV-infected and HCQ/Rapa-treated eyes was quantified using ImageJ analysis. The data represent mean ± SD from *n* = 4-6 mice per group. *P  <  0.05; **P  <  0.005; one-way ANOVA; C vs. ZIKV and ZIKV vs. drugs. (**F**) In another set of experiments, ZIKV (Z) and HCQ/Rapa-treated mice’s anterior segment tissue lysates were subjected to immunoblotting for autophagy marker LC3B. (**G**) Densitometric analysis of LCB-II/I ratio (normalized to β-actin) was performed using ImageJ. Each data point represents mean ± SD from two pooled eyes from three biological replicates (*n* = 6 eyes/group). **P  <  0.005; ****P  <  0.0001; one-way ANOVA; C vs. treatment.

Based on our above *in vitro* findings, we established that ZIKV induces autophagy in TM and blocks autolysosomal maturation to avoid viral degradation. Moreover, autophagic activation in TM supports viral replication, while autophagy inhibition limits viral replication. To investigate the physiological relevance of autophagic activity in an *in vivo* model of ZIKV ocular infection, we examined whether ZIKV induces autophagy in the ocular milieu and whether autophagic modulation plays any role in ZIKV-induced ocular complications. To assess this, we used our previously established mouse model of ZIKV-induced trabecular infection (10). We infected IFNAR1^-/-^ mice with ZIKV via anterior chamber inoculation and evaluated the levels of LC3B in the anterior segment tissue (7 days post-infection) by immunoblotting. Similar to our *in vitro* human TM cell response, ZIKV infection showed an increased accumulation of lipidated LC3B-II in mouse anterior segment tissue (Fig. 6A and B), indicating autophagy activation in the anterior segment of the eye.

Next, we used the Ad5-mRFP-GFP-LC3 expression construct to assess the autophagic flux activity in the eye upon ZIKV infection. Ad5-mRFP-GFP-LC3 expression vector was intravitreally injected into IFNAR1^-/-^ mice’s eyes 3 days prior to ZIKV infection. Eyes injected with Ad5-mRFP-GFP-LC3 without ZIKV challenge were used as a control. Consistent with our *in vitro* findings, we found an increased accumulation of GFP^+^/RFP-GFP^+^LC3 autophagosomes in the mouse anterior segment, especially in the TM region in ZIKV-infected (7 days post-infection) animals as compared to controls (Fig. 6C), suggesting ZIKV blocks autophagic flux, resulting in the accumulation of autophagosomes.

To further determine the role of autophagy in ZIKV-induced ocular complication, we intraperitoneally treated IFNAR1^-/-^ mice with HCQ (40 mg/kg of body weight, once daily for four consecutive days) to block autophagy and rapamycin (1 mg/kg of body weight, once daily for four consecutive days) to activate autophagy as per earlier reports (19, 37). These mice were challenged with ZIKV (1 × 10^4^ PFUs) through anterior chamber inoculation 1 day post-HCQ/rapamycin treatment (treatments continued for three consecutive days post-ZIKV infection). The ocular pathology in treated vs untreated groups was assessed using Micron IV fundus imaging. Like our previous observation, the ZIKV-infected mice displayed chorioretinal atrophy in the eye in comparison to uninfected controls. Surprisingly, the autophagy inhibitor HCQ treatment alleviated the ZIKV-induced chorioretinal atrophy, as opposed to the autophagy inducer rapamycin, which further aggravated the retinal lesions (Fig. 6D and E). To confirm the HCQ and rapamycin treatment on autophagic activity in the ocular milieu, we assessed the anterior segment tissue for autophagy marker protein LC3B lipidation. Our western blot analysis confirmed the accumulation of LC3B-II in infected and respective drug-treated samples, indicating autophagic activity in ocular tissue (Fig. 6F and G). Collectively, these findings suggest that ZIKV infection induces autophagy in ocular tissue and perturbs autophagic flux as a means for their egress, and autophagy modulation can be harnessed to combat ZIKV-induced ocular complications.

### Autophagy modulation by HCQ modulates ZIKV-altered host transcriptome in TM cells

**Fig 7:**
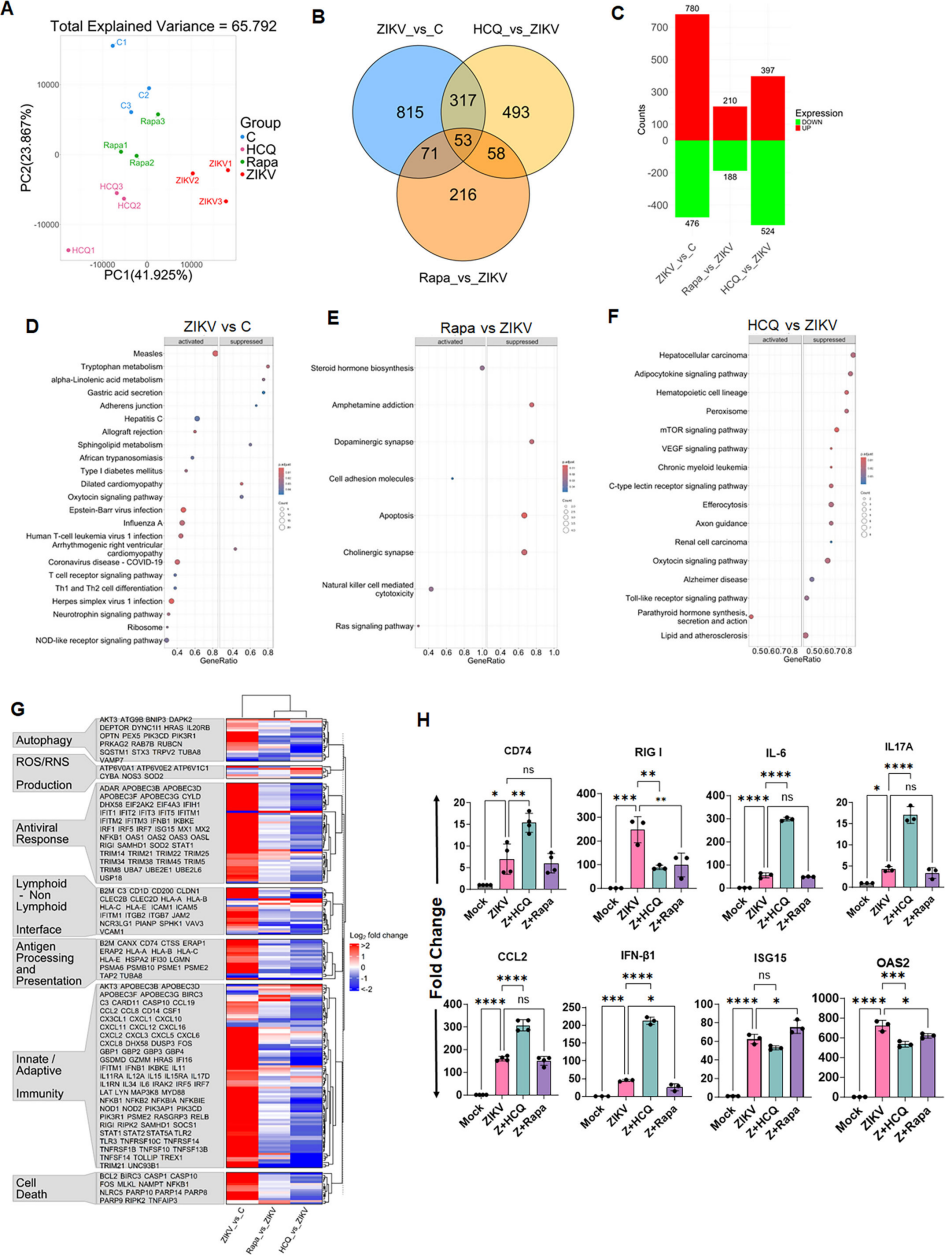
Autophagy modulation modulates ZIKV-altered host transcriptome in TM cells. HTMCs (*n* = 3) were infected with ZIKV (multiplicity of infection [MOI]: 1) in the presence and absence of rapamycin (Rapa) and HCQ for 48 h. Mock-treated cells were used as a control (C). The total RNA from infected/treated HTMCs was subjected to RNA-seq analysis. (**A**) Principal-component analysis (PCA) of RNA-seq data from three biological replicates showing PC1 vs. PC2 plot. (**B**) The Venn diagram indicates shared DEGs between mock (C), ZIKV, HCQ, and Rapa-treated groups. (**C**) Waterfall plot representing the total number of upregulated and downregulated genes in ZIKV-infected and HCQ/Rapa-treated groups. (**D–F**) GSEA for KEGG pathways for DEGs between ZIKV vs. control (**D**), Rapamycin vs. ZIKV (**E**), and HCQ vs ZIKV (**F**). (**G**) Heatmap reflecting the log2 fold change of a known list of genes involved in critical biological pathways involving autophagy, ROS/RNS production, anti-viral immune response, innate and adaptive immunity, antigen processing and presentation, cell adhesion/junction, and cell death pathways. (**H**) Total RNA from ZIKV-infected and HCQ and rapamycin-treated cells was subjected to qPCR for the indicated gene expression. The data represent mean ± SD from three biological replicates. *P  <  0.05; **P  <  0.005; ***P  <  0.0005; ****P  <  0.0001; one-way ANOVA; C vs. treatment.

Our *in vitro* and *in vivo* findings assert that ZIKV exploits autophagy in TM for its replication, and autophagic modulation not only regulates viral transmission but also attenuates ZIKV-induced ocular complications. Next, to attain a global perspective of transcriptomic changes induced by ZIKV in TM and how autophagy modulation regulates these cellular changes, we performed RNA sequencing (RNA-seq) studies. We infected HTMC with ZIKV for 48 h in the presence and absence of HCQ and rapamycin and performed RNA-based transcriptional profiling to characterize gene expression profiles. Our Principal Component Analysis (PCA) showed a significant variation among biological replicates (Fig. 7A), resulting in different baseline signatures. We observed that, on a global transcriptional level, gene expression signatures differed drastically between ZIKV-infected and HCQ-, rapamycin-treated samples with a total of 2,023 genes showing differentially dysregulated (q-value ≤0.05 and absolute log_2_ fold change >1), among which only 53 genes were commonly dysregulated between all three groups (Fig. 7B). Among these differentially expressed genes (DEGs), 815 genes were uniquely dysregulated in ZIKV-infected HTMC in comparison to uninfected mock controls, whereas HCQ and rapamycin treatment showed unique dysregulation of 493 and 216 genes, respectively, compared to ZIKV-infected cells (Fig. 7B). In addition, among these DEGs, a total of 780, 210, and 397 genes showed upregulation, and 476, 188, and 524 genes showed downregulation in ZIKV-infected, rapamycin- and HCQ-treated groups, respectively (Fig. 7C). Gene set enrichment analysis (GSEA) for KEGG pathways revealed significant alterations in pathways associated with viral infectivity, lipid/amino acid metabolism, inflammation, and cell death in ZIKV-infected HTMC. On the contrary, GSEA analysis from HCQ-treated groups showed significant suppression of many of these pathways, including lipid metabolism and TLR-mediated inflammatory signaling pathways (Fig. 7D through F). A heatmap was constructed to visualize the significant dysregulation of genes with critical biological functions, including innate and adaptive immunity, anti-viral immune response, antigen processing and presentation, cell death pathways, reactive oxygen and nitrogen species production, cell adhesion/junctions, and autophagy (Fig. 7G). Our heatmap analysis revealed significant dysregulation of multiple genes involved in canonical anti-viral (e.g., ISG15, MX1, MX2, OAS1, OAS2, OAS3, IFNB1, RIGI, STAT1, IRF5, IRF7, IFITM1, IFITM2, IFITM3, TRIMs), innate/adaptive immunity (e.g., TLR3, RIGI, MyD88, NFKB1, CCL2, CCL8, CCL19, CXCL1, CXCL2, CXCL3, CXCL5, CXCL6, CXCL8, CXCL10, CXCL11, CXCL12, CXCL16, multiple interleukins, TNFs), antigen processing and presentations (e.g., HLAs, PSMEs, CD74, ERAP1 & ERAP2), cell death (BCL2, CASP 1 & 10, RIPK2, MLKL, NAMPT, NLRC5, GSDMD, PARP8, PARP9, PARP10, PARP14), cell junction/adhesion (e.g., ICAM1, ICAM5, VCAM1, VAV3, SPKH1, JAM2), and autophagy (e.g., AKT3, DEPTOR, OPTN, PEX5, RAB7, STX3, TRPV2, SQSTM1, ATG9, BNIP3) pathways in ZIKV-infected HTMC (Fig. 7G). Rapamycin treatment moderately downregulated some of these pathways, whereas HCQ treatment robustly modulated these host-mediated responses, indicating a cell-protective ability of HCQ. To further validate the effect of autophagy modulation on host-mediated defense mechanisms, we performed qPCR from ZIKV-infected HTMCs in the presence and absence of HCQ and rapamycin. Our results show that HCQ treatment significantly induced the expression of innate immune/anti-viral mediators (e.g., CD74, IL-6, IL17A, CCL2, and IFN-β1) (Fig. 7H). We observed a reduction in ZIKV-induced PRR and some ISGs, for example, ISG15 and OAS2, suggesting HCQ may inhibit PRR-mediated signaling, which plausibly resulted in reduced expression of ISGs. These results are consistent with previous findings where HCQ has been shown to suppress PRRs, ISGs, and virus-induced host response (38–40). The HCQ and rapamycin treatment alone (without ZIKV) at the given concentration has marginal or no effect on the abovementioned HTMC transcripts (Fig. S7). Collectively, our RNA-seq and qPCR findings confirm that autophagy modulation remodels the ZIKV-altered host defense mechanisms in TM cells.

## DISCUSSION

ZIKV is a re-emerging RNA virus known to cause significant neuro-ophthalmological complications, developmental delays, cognitive dysfunctions, and impaired fertility (10, 11, 41). During the 2015 epidemics in the Americas, ZIKV was reported to cause severe ocular manifestations such as unilateral microcornea, iris coloboma, chorioretinal atrophy, hypoplasia, focal pigmented mottling, RPE mottling, retinal focal spots, severe retinal vessel attenuation, macular pigmentation alterations including pallor, optic nerve atrophy, optic disc anomalies, and congenital glaucoma in infants born to ZIKV-infected mothers (3–5, 7, 41). Our recent investigation revealed that ZIKV can invade and replicate in TM and cause trabeculitis in mice (10). However, the cellular mechanism of ZIKV replication in TM is unknown.

Viruses are known to impair autophagy and utilize lipid-rich autophagic compartments for their replication and egress (20, 30). ZIKV has been shown to induce autophagy in different cell types, such as JEG3 cells and tissue from the placental lining (19), brain neural stem cells (21), and skin fibroblasts (42), which is suggested to provide the replication advantage to the virus. Further evidence for the proviral role of autophagy stems from studies in ATG16 hypomorphic (HM) (*Atg16l1*^HM^) pregnant mice that exhibited decreased autophagic activity with reduced ZIKV infection in HM placentas and low fetal ZIKV titers as compared to wild-type (WT) controls (19). In this study, we investigated the role of autophagy in ZIKV TM infectivity using human trabecular meshwork cells (HTMC) *in vitro* and a mouse model of ocular ZIKV infection *in vivo*. TM plays a crucial role in maintaining intraocular pressure. Damage or dysfunction to TM cells is known to cause IOP elevation, leading to glaucoma (10, 43). Recently, we observed that ZIKV induces the expression of multiple TAM receptors (AXL, TYRO3, and MERTK) known for ZIKV entry (9), in HTMC. The induced expression of these receptors in HTMC by ZIKV could be a potential mechanism for viral entry in TM and associated pathology. In consonance with this finding, our current data indicate that ZIKV is highly permissive to HTMC, which correlates with the pathological consequences of ZIKV infection in TM. Since ZIKV demonstrated enhanced susceptibility toward HTMC, we presumed that it might deploy an innate inflammatory and anti-viral immune response to contain the viral infection. Our findings suggest an early induction of anti-viral response by ZIKV in HTMC, which is later replaced by copious amounts of acute inflammatory cytokines (IL-6, IL-1β, and TNF-α) alongside type II interferon IFNγ, indicating overt and dysregulated immune response due to uncontrolled ZIKV replication in TM, coinciding with extensive cell death. Overt production of inflammatory cytokines (IL-6, IL-1β, and TNF-α) has been associated with exacerbated infection with SARS-CoV/MERS viruses previously (44). In fact, TLR3-mediated production of IL-6 has been demonstrated to be responsible for reduced interferon production and downregulated anti-viral responses in human neural progenitor cells (hNPCs) and iPSC-derived astrocytes upon ZIKV infection (45).

Viral infection and tissue destruction can set the stage for autophagy activation. Multiple Flaviruses, including ZIKV and DENV, have been shown to exploit autophagy for their replication and modulate the host immune response (29, 42). In this study, we investigated the role of autophagy in ZIKV TM infectivity, intending to define its contribution to ZIKV-induced ocular pathology. Our data demonstrated that ZIKV infection triggers autophagy activation in HTMC, leading to increased autophagosome accumulation. These results are concordant with previous findings where ZIKV infection has been shown to activate autophagy in different cell types and associated tissues (19, 21, 42). In degradative autophagy, increased accumulation of autophagosomes initiates the clearance of cellular cargo via the fusion of autophagosomes with autolysosomes. However, several viruses are known to block the autophagic flux to prevent autolysosomal maturation and escape autophagic degradation (16). To uncover the role of ZIKV in regulating the autophagic flux in TM, we used the Ad5-mRFP-GFP-LC3 construct and observed an increased accumulation of autophagosomes over autolysosomes in HTMC and mouse anterior segment (AS) tissue, indicating an impaired autophagic flux. From our findings, we conclude that the accumulation of autophagosomes in ZIKV-infected TM cells or mouse AS tissue resulted from both activation of autophagosome formation as well as suppression of autophagosome-lysosome fusion. Consistent with our findings, the blockade of the maturation of autophagosomes into autolysosomes has been demonstrated during SARS-CoV-2 infection of different cell types, with a major role of its accessory protein ORF3 (32, 46).

Following the observation that ZIKV impairs autophagic flux to block autolysosomal maturation, we reasoned that ZIKV might use intermediate autophagic compartments for its replication and spread. To assess this, we next tracked the ZIKV cellular abode along the autophagic compartments using the TEM and co-immunolocalization study. Our findings indicate the presence of ZIKV in autophagosomes alongwith a significant co-localization of viral dsRNA intermediates and ZIKV-E antigen (4G2) in late-endosomes/lysosomes, confirming its ability to replicate in these subcellular compartments. To our knowledge, this is the first instance that highlights the unusual ability of ZIKV to survive in late-endosomal-lysosomal compartments of HTMCs. Our finding is consistent with an observation where a β-coronavirus, MHV, has been shown to use lysosomes for its egress (30). Our observation of endosomal-lysosomal localization of ZIKV with blockade of autophagic flux upon infection progression was further corroborated by a time-dependent accumulation/degradation of autophagy marker proteins LC3B-II and SQSTM1/p62 with a concomitant increase in organelle proteins Rab5/Rab7 (endosomes), and LAMP1 (lysosomes). To further confirm the blockade of autolysosomal maturation, we investigated VPS39, a core component of the HOPS complex, and STX17, a SNARE complex constituent, proteins that mediate autophagosome fusion with lysosomes, leading to the formation of mature autolysosomes. VPS39 is a HOPS-specific subunit that interacts with Rab7 during lysosomal fusion and forms a complex with STX17, a subunit of the SNARE complex. The STX17 SNARE complex has been shown to translocate to autophagosomes when they are close and disappear following autolysosomal maturation within 10 minutes (47). In our study, we do not see degradation or reduced expression of VPS39 or STX17 molecules expressed on autophagosomes/amphisomes, which corroborates the notion that ZIKV infection in TM indeed blocks autophagosomes/amphisomes fusion with lysosomes without affecting their numbers or biogenesis. Consistent with our results, a few recent studies have also demonstrated the blockade of autophagic flux by SARS-CoV-2 and its accessory protein ORF3a by inhibiting the autophagosome-lysosome fusion via interrupting the Rab7-VPS39 interaction or causing failed SNARE complex formation essential for autolysosome formation (31, 32, 46). Similarly, ZIKV NS3 protein has been shown to interact with HOPS complex, and NS3-NS2B protease interaction with HOPS/CORVET is known to inhibit late endosome-lysosome fusion (34).

Autophagy is a multi-stage dynamic process, and the precise mechanistic details on which stages of autophagy could be beneficial for ZIKV replication in TM are unclear. Therefore, we used a pharmacological activation and inhibition approach to determine the relationship between autophagic events and ZIKV infectivity. Our findings revealed a significant abrogation of ZIKV replication in HTMC by treatment with Baf-A1 and HCQ that interfere with autolysosomal fusion or autophagic maturation. Surprisingly, 3-MA, an early-stage autophagy inhibitor, inhibited the ZIKV replication to a lesser extent than Baf-A1 or HCQ, both of which impede autophagy at the late lysosomal stage and impair lysosomal activity. These findings suggest that ZIKV utilizes late-stage autophagic vesicles as its replication niche and has evolved to shield itself from lysosomal killing. The fact that ZIKV exploits autophagy for its survival was further supported by dose-dependent enhancement in ZIKV replication with rapamycin/torin-1 treatment. Our findings corroborated with several other studies where autophagic inhibition with Baf-A1, HCQ, and 3-MA has shown reduced viral burden in different experimental models, including ZIKV and SARS-CoV-2 (19, 21, 33).

We further tested the role of autophagy in ZIKV replication in the ocular milieu using our previously established mouse model (10). In our IFNAR1^-/-^ mouse model, we demonstrated that ZIKV induces autophagy in the anterior segment tissue and blocks the autophagic flux, as indicated by the increased accumulation of GFP^+^RFP^+^ autophagosomes over RFP^+^ autolysosomes in TM/Schlemm’s canal region. Furthermore, treatment of ZIKV-infected animals with an autophagy inhibitor and an FDA-approved drug HCQ remarkably alleviated the ZIKV-induced chorioretinal atrophy and retinal scarring compared to ZIKV-infected/untreated control mice. By contrast, autophagy induction by rapamycin treatment aggravated the virus-associated retinal atrophy. These findings further strengthen the proposition that ZIKV activates autophagic activity in ocular tissue for its egress, and autophagic inhibition at autolysosomal maturation could be a beneficial strategy to prevent or treat ZIKV-induced ocular complications. Our study corroborated findings where autophagy inhibition using HCQ has been shown to limit vertical transmission of ZIKV in pregnant mice (19) and improve SARS-CoV-2-induced lung lesions and pneumonia (33). Autophagy is known to be modulated in multiple eye diseases and acts differently in different disease conditions (48). Our findings could be applicable to other ocular microbial pathogens that use autophagy as their survival mechanism. In line with our aim to develop autophagy inhibitors as antivirals for ocular viral infections, we recently developed multiple novel small-molecule quinoline derivatives (HCQ analogs) that showed anti-ZIKV activities in different ocular cells, including HTMC and retinal pigmented epithelium (38).

ZIKV has been shown to cause transcriptomic alteration in various cellular models, including astrocytes, Sertoli cells, and retinal pigmented epithelium (49–51). To depict the role of ZIKV infection on the TM cell transcriptome and how autophagy modulation regulates these cellular changes in TM, we performed RNA-seq analysis. Our data revealed that ZIKV altered several canonical pathways with key biological functions in TM, including innate/adaptive immunity, anti-viral response, antigen presentation and processing, cell junction/adhesion, cell death, and autophagy pathways. Interestingly, autophagy modulation by HCQ attenuated cellular changes in TM by suppressing these ZIKV-induced pathways. We observed that HCQ treatment induced the innate cytokine response and inhibited the PRRs that sense viral RNA, with subsequently reduced expression of ISGs. Our study is supported by findings where HCQ has been shown to suppress antigen presentation and interferon signaling in several autoimmune and inflammatory diseases and during viral infections (38–40).

In summary, our study provides comprehensive evidence on the proviral role played by autophagy in ZIKV infectivity in the human trabecular meshwork and mouse ocular milieu. Using a combination of *in vitro* and *in vivo* studies, our findings confirmed that autophagy, despite being a host protective mechanism, favors ZIKV replication in the ocular milieu. Thus, careful manipulation of autophagy can be opted as a therapeutic means to minimize ZIKV-associated ocular complications. Finally, our study provides a foundation for further research into virus-ocular interaction and developing suitable modalities targeting autophagy as potential anti-viral theraputic avenue.

## MATERIALS AND METHODS

### Antibodies and reagents

Antibodies used in this study were purchased from the following sources: NS3 (GeneTex, GTX133309), 4G2 (GeneTex, GTX57154), dsRNA (Millipore Sigma, MABE1134), Rab7 (Santa Cruz, sc-376362), and VPS39 (Protein Tech, 16219-1-AP). LC3B (2775S), SQSTM1/p62 (39749S), Rab5 (2143S), STX17 (31261S), and LAMP1 (9091S) antibodies were purchased from Cell Signaling Technology. Pharmacological activators/inhibitors chloroquine (14194), hydroxychloroquine (17911), bafilomycin A1 (11038), rapamycin (13346), and torin 1 (10997) were purchased from Cayman Chemicals (Ann Arbor, MI).

### Cells and culture conditions

Primary human trabecular meshwork cells (Pr. HTMC) (10, 52) that were originally obtained from Dr. Gulab Zode (Professor, UC Irvine) and characterized per consensus recommendations for TM cell culture and characterization (53). HTMCs were cultured and maintained in Dulbecco’s minimal essential medium (DMEM), supplemented with 10% fetal bovine serum (FBS), 10 µg/mL L-glutamine, 1× penicillin-streptomycin solution at 37°C in a CO_2_ (5%) incubator with 95% humidity. Vero E6 cells (ATCC CRL-1586) were cultured in DMEM medium containing 10 µg/mL L-glutamine and 1× penicillin-streptomycin (P/S) supplemented with 10% FBS. For virus propagation, Aedes albopictus, C6/36 cells (ATCC CRL-1660) were cultured in Eagle’s Minimum Essential Medium (EMEM) containing 10 µg/mL L-glutamine and 1× penicillin-streptomycin supplemented with 10% FBS at 30°C in a CO_2_ incubator.

### Mice and ethics statement

IFNAR1 knockout (IFNAR1^-/-^) mice were originally purchased from Jackson Laboratories and bred in-house. Both male and female mice aged 6–10 weeks were used for this study. All animals were housed in a controlled access, the Association for Assessment and Accreditation of Laboratory Animal Care (AAALAC) accredited, Office of Animal Resources (OAR) facility, maintained in a 12:12 h light/ dark cycle, and fed on lab diet rodent chow (Labdiet Pico Laboratory, Saint Louis, MO) and water *ad libitum*. Mice were treated in compliance with the Association for Research in Vision and Ophthalmology (ARVO) Statement for the Use of Animals in Ophthalmic and Vision Research. All biohazard and animal procedures were approved by the Institutional Biosafety Committee (IBC) and Animal Care and Use Committee (ACUC) of the University of Missouri, Columbia.

### Zika virus strain and infection procedure

The ZIKV strain PRVABC59 (NR-50240), which was initially isolated from human blood in Puerto Rico in December 2015, was obtained from BEI Resources, National Institute of Allergy and Infectious Diseases (NIAID), and propagated in Aedes albopictus, C6/36 cells. The viral titers were determined using the plaque assay. Small aliquots were prepared and stored in a −80°C freezer for infection studies.

For *in vitro* experiments, cells were challenged with ZIKV at a multiplicity of infection (MOI) of 1. For pharmacological inhibition/activation studies, cells were pretreated with respective drugs for 1 h, followed by virus adsorption in serum-free media for 1 h. After adsorption, cells were replenished with media containing drugs and incubated until the desired endpoint. For i*n vivo* experiments, animals were infected with 1 × 10^4^ PFU of ZIKV via anterior chamber injections.

### Plaque assay

ZIKV plaque assay was performed using a protocol we described previously (38). Briefly, a confluent monolayer of Vero cells was infected with serial dilutions of ZIKV stock culture. One hour following viral adsorption, the viral inoculum was removed, and the cell monolayer was overlayed with the first overlay media containing a 1:1 mixture of 2× EMEM, 4% FBS, 2× P/S, 20 mM MgCl_2_, and 1.6% Noble Agar. The next day, a second overlay media containing DMEM, 1 mg/mL BSA, 40 mM MgCl_2_, 0.2% glucose, 2 mM sodium pyruvate, 4 mM L-glutamine, 4 mM oxaloacetic acid, 1× P/S, and 0.1% sodium bicarbonate was added. The plates were incubated in a CO_2_ incubator at 37°C for 5 days. Following incubation, cells were fixed with 10% tricarboxylic acid (TCA) for 20 min, and the agar overlay was removed gently without disturbing the cell monolayer. Viral plaques were stained with 0.2% crystal violet for 20 min, followed by a wash with MilliQ water. The plaques were counted, and titers were estimated as log_10_ PFU/mL.

### Cytotoxicity assay

The cytotoxicity of different autophagic modulators (rapamycin, torin-1, 3-MA, Baf-A1, and HCQ) on HTMC was determined using Cell Proliferation Reagent WST-1 per manufacturer’s instructions (Cat # 5015944001, Millipore-Sigma, St. Louis, MO)

### Immunofluorescence assay

For immunofluorescence assay (IFA), cells were seeded in a Nunc four-well chamber slide (Fisher Scientific, Rochester, NY) and infected with ZIKV at an MOI of 1 at ~70%–80% confluency. Untreated cells were used as mock controls. At desired endpoints, cells were fixed using 4% paraformaldehyde in 1× PBS overnight at 4°C. After three washes with 1× PBS, cells were blocked and permeabilized using 2% (wt/vol) BSA with 0.4% (vol/vol) Triton X-100 made in PBS (blocking buffer) for 1 h at room temperature (RT) in a humidified chamber. Subsequently, the cells were incubated with the primary mouse/rabbit (anti-E flavivirus group antigen [4G2] and/or anti-LC3B) antibodies in the blocking buffer (1:100 dilution) overnight at 4°C in a humidified chamber. Following primary antibody incubation, cells were washed three times with 1× PBS and incubated with anti-mouse/rabbit Alexa Fluor 488/594-conjugated secondary antibodies (1:200 dilutions) for 1 h at RT. After incubation, cells were washed four times with 1× PBS and mounted in Vectashield anti-fade mounting medium containing DAPI (Vector Laboratories, Burlingame, CA). The slides were imaged using a Keyence (Keyence, Itasca, IL) or Leica SP8 (Leica, Deerfield, IL) fluorescence microscope.

### Western blotting

Western blot was performed using a protocol we described previously (52). Briefly, cells were lysed using RIPA lysis buffer with a Halt protease and phosphatase inhibitor cocktail (Thermo Scientific, Rockford, IL). The total protein amount was estimated using the BCA method per manufacturer’s instructions (Thermo Scientific, Rockford, IL). Thirty microgram of total protein was resolved on a 4%–20% gradient sodium dodecyl sulfate-polyacrylamide gel electrophoresis (SDS-PAGE) gel and transferred onto nitrocellulose or PVDF membranes. Following transfer, the membrane was blocked using 5% non-fat skim milk and then washed with 1× TBST (Tris-glycine buffer containing 0.5% Tween 20). The blots were incubated with primary antibodies (1:1,000 dilutions) diluted in 3% BSA overnight at 4°C with gentle agitation. After incubation with the primary antibodies, the membranes were washed three times using 1× TBST, followed by incubation with anti-mouse/rabbit HRP-conjugated secondary antibodies (1:2,000 dilutions) at RT for 2 h. Subsequently, after three washes with 1× TBST, the blots were developed using Supersignal West Femto chemiluminescent substrate and imaged using iBright FL1500 imager (Thermo Fisher Scientific, Rockford, IL).

### RNA isolation and quantitative PCR

Total cellular RNA was extracted from treated/untreated cells using TRIzol reagent per the manufacturer’s instructions (Thermo Scientific, Rockford, IL). RNA (1 µg) was reverse transcribed to synthesize cDNA using a Maxima first-strand cDNA synthesis kit, per the manufacturer’s instructions (Thermo Scientific, Rockford, IL). The cDNA was amplified using human gene-specific PCR primers in a 96-well plate using QuantStudio 3 Real-Time PCR system (ThermoFisher Scientific, Rockford, IL). The relative expression of the gene of interest was normalized in proportion to the constitutive gene 18s RNA as an internal control and quantitatively analyzed using the 2^−ΔΔCt^ method and represented as fold change expression.

### Autophagy activation/flux measurement

The autophagic flux in ZIKV-infected cells/tissue was measured using two different assays. In one assay, an adenovirus vector expressing the mRFP-GFP-LC3 construct was used. This assay relies on the principle that GFP fluorescence (pKa: 5.9) is quenched in acidic compartments, whereas RFP fluorescence (pKa: 4.5) is stable in acidic compartments such as lysosomes. The mRFP-GFP-LC3 exhibits both red and green (yellow in merged) fluorescence, indicating autophagosomes. Expression of RFP alone indicates autolysosomes. Measuring red/yellow-green puncta per cell represents the rate of autophagic flux. For this assay, primary HTMC cells were seeded in Nunc four-well chamber slide (Fisher Scientific, Rochester, NY) and transduced with Ad5-mRFP-GFP-LC3 vector for 24 h, followed by ZIKV infection for an additional 24 h. Ad5-mRFP-GFP-LC3-transduced cells without ZIKV infection were used as a mock control. Following incubation, cells were briefly (for 5 min) fixed with 2% paraformaldehyde, and fluorescent images were immediately taken using a Keyence fluorescence microscope (Keyence, Itasca, IL). The RFP^+^/GFP^+^ puncta were quantified from multiple frames using ImageJ. For *in vivo* studies, the Ad5-mRFP-GFP-LC3 vector was injected via intravitreal injections, followed by ZIKV infection by anterior chamber inoculation. 72 h following infection, the eyes were enucleated and fixed in OCT medium. Eyes cryosections were observed and imaged for RFP/GFP positivity using a Keyence fluorescence microscope.

In another assay, autophagy flux in ZIKV-infected Pr. HTMC was measured using CYTO-ID autophagy detection kit (ENZ-51031-0050) per the manufacturer’s instructions (Enzo Life Sciences, Farmingdale, NY). This assay uses a cationic amphiphilic tracer (CAT) probe that rapidly partitions into cells with induced phospholipidosis and enables the labeling of vacuoles associated with autophagic pathways.

### Transmission electron microscopy

For TEM analysis, HTMCs were cultured in a 100 mm petri dish and infected with ZIKV at an MOI of 1 for 48 h. Following infection, cells were harvested and fixed with a fixative containing 2% paraformaldehyde and 2% glutaraldehyde in 100 mM sodium cacodylate buffer (pH 7.35, Sigma Aldrich, St. Louis, MO). After fixation, the cell pellets were embedded in HistoGel (Thermo Scientific, Kalamazoo, MI). The HistoGel-embedded pellets were rinsed with a buffer containing 100 mM sodium cacodylate and 130 mM sucrose (pH 7.35). Secondary fixation was performed using 1% osmium tetroxide (Ted Pella, Inc., Redding, CA) at 4°C for 1 hour. After fixation, samples were rinsed first with cacodylate buffer and then with distilled water. En bloc staining was carried out with 1% aqueous uranyl acetate at 4°C overnight, followed by rinses with distilled water. Dehydration was performed using a graded ethanol series (20%, 50%, 70%, 90%, and 100% for three times), followed by a transition to acetone. Specimens were then infiltrated with Epon resin and polymerized at 60°C for 48 hours. Ultrathin sections (75 nm) were cut using an ultramicrotome (UC7, Leica Microsystems, Germany) and a diamond knife (Diatome, Hatfield, PA). Thin sections were post-stained with UranyLess and 3% lead citrate. Imaging was performed with a JEOL JEM-1400 transmission electron microscope (JEOL, Peabody, MA) operating at 120 kV, equipped with a Gatan Ultrascan 1000 CCD camera (Gatan, Inc., Pleasanton, CA).

### RNA sequencing and analysis

For RNA sequencing, total RNA was extracted from ZIKV-infected and HCQ- and rapamycin-treated HTMC in biological triplicates using RNeasy Mini Kit (Qiagen, Hilden, Germany). RNA sequencing (RNA-seq) libraries were constructed from high-quality RNA using NEBNext Ultra II RNA Library Prep kits for Illumina (New England BioLabs, Ipswich, MA) per the manufacturer’s instructions. Sequencing was performed on the Novaseq 6000 platform as paired-end 150 bp reads (Illumina), with a sequencing depth of approximately 50 million reads per sample. The sequencing was performed by Novogene Corporation Inc. (Sacramento, CA).

The raw RNA-seq reads quality check was performed by using FastQC (Version 0.12.1) (54) and aggregated through the MultiQC tool (Version 1.17) (55). The reads were later trimmed with Cutadapt (Version 4.6) (56), for removing Illumina adapters, ambiguous nucleotides (N’s), any paired sequence reads with read lengths <20 bp, and reads with a quality Phred Score of less than 20. The trimmed reads were aligned to the reference human genome (GRCh38.111) from Ensembl using HISAT2 (Version 2.2.1) (57) to achieve a high overall alignment (~96.7%). The aligned files were converted to sorted bam files using Samtools (Version 1.18) (58), and further processed with Cufflinks (Version 2.2.1) (59) for generating gene expression abundance levels. Differential gene expression analysis (DGEA) was carried out using the Cuffdiff method, which is available through the Cufflinks package. Further analysis was conducted using R (60) and its packages. GSEA was done through the cluster Profiler (61) package, while PCA, Venn, and Heatmap were made using stats, ggvenn (62), and ComplexHeatmap (63) packages, respectively, and graphs were drawn using ggplot2 (64).

### Statistical analysis

The statistical differences between experimental groups were analyzed using GraphPad Prism 10 V10.1.2 (GraphPad Software, La Jolla, CA). The one-way analysis of variance (ANOVA), two-way ANOVA, or unpaired t-test was used to compare the significance level between experimental groups, as indicated in figure legends. A value of *P* < 0.05 was considered statistically significant. All data are expressed as the means ± standard deviation (SD) from three biological replicates unless indicated otherwise.

## Data Availability

All relevant data are within the manuscript and figures. The transcriptomics (RNAseq) data were deposited in the GEO database with accession ID GSE270987.

## SUPPLEMENTAL MATERIAL

Supplemental figures spectrum.01034-25-s0001.docx Fig. S1 to S7.

ASM does not own the copyrights to Supplemental Material that may be linked to, or accessed through, an article. The authors have granted ASM a non-exclusive, world-wide license to publish the Supplemental Material files. Please contact the corresponding author directly for reuse.
