# Supplementary material for: Zika virus impairs autophagic flux in trabecular meshwork, and inhibition of autophagy restricts ocular viral transmission and associated pathology
Source: Microbiol Spectr. 2025 Sep 8;13(10):e01034-25. doi: 10.1128/spectrum.01034-25 (PMC12502531; doi:10.1128/spectrum.01034-25)
Supplement: Supplemental figures — Fig. S1 to S7. [file spectrum.01034-25-s0001.docx]

**Zika virus impairs autophagic flux in trabecular meshwork, and inhibition of autophagy restricts ocular viral transmission and associated pathology.**

**Authors:** Faraz Ahmad^1,†^, Prince Kumar^1,†^, Pallav Singh^2^, Trupti Joshi^2,3,4^, Pawan Kumar Singh^1, #^

^†^ These authors contributed equally.

**Affiliations:**

^1^Department of Ophthalmology, Mason Eye Institute, University of Missouri School of Medicine, Columbia, Missouri, USA.

^2^MU Institute of Data Science and Informatics, University of Missouri, Columbia, Missouri, USA

^3^Department of Biomedical Informatics, Biostatistics and Medical Epidemiology, University of Missouri, Columbia, Missouri, USA.

^4^Christopher S Bond Life Sciences Center, University of Missouri, Columbia, Missouri, USA

**Running Head:** ZIKV exploits autophagy in TM.

**# Address Correspondence to:**

**Pawan Kumar Singh, Ph.D.**

Department of Ophthalmology, Mason Eye Institute

University of Missouri School of Medicine

1 Hospital Dr, Columbia, Missouri, 65212, USA.

Email: [pksfcq@health.missouri.edu](mailto:pksfcq@health.missouri.edu)

**Supplementary Figures**:


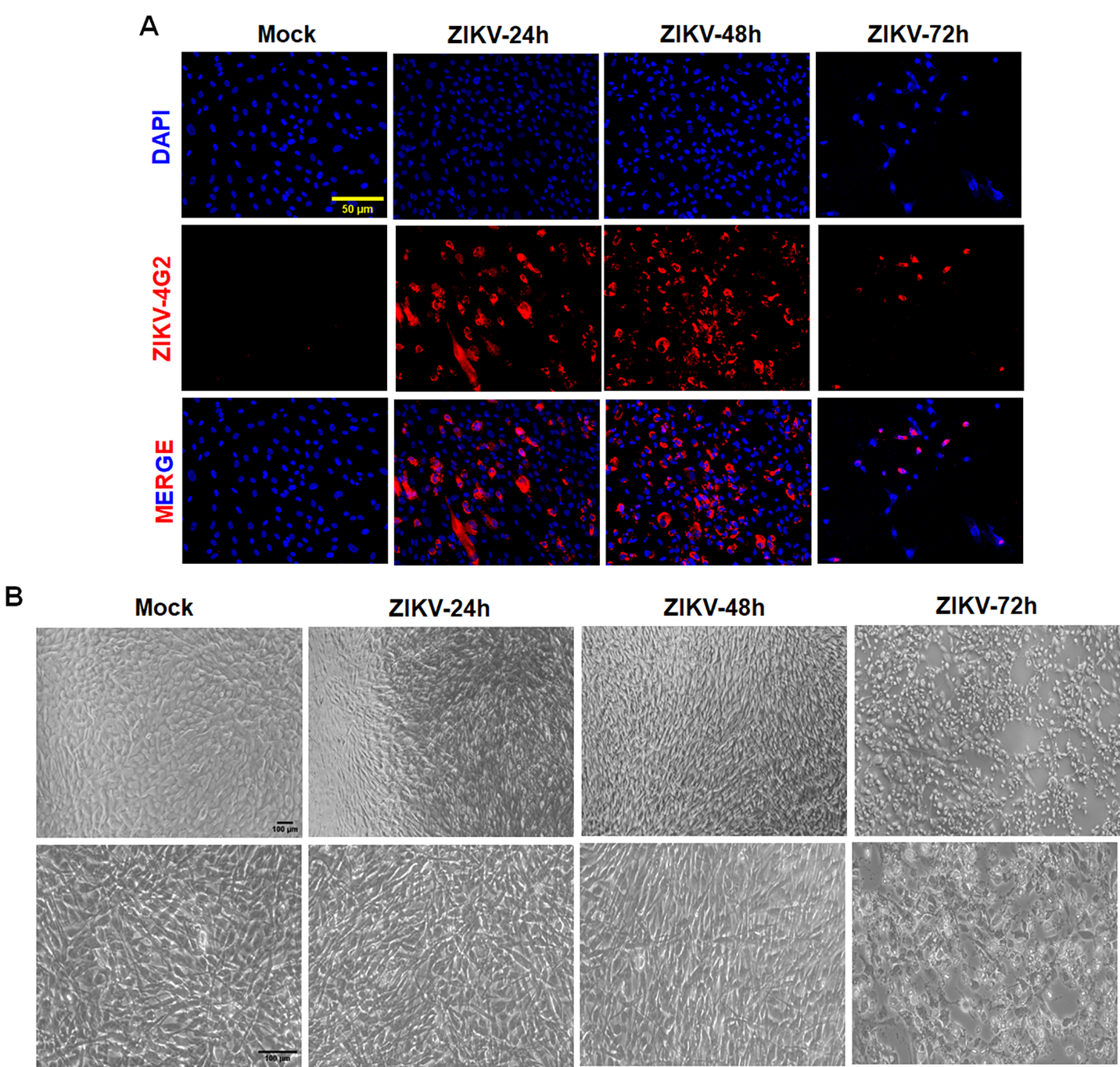


**Supplementary Figure 1**: **ZIKV permissively infects HTMCs. (A)** Primary Human Trabecular Meshwork Cells (HTMCs) were infected with ZIKV strain PRVABC59 at an MOI 1 for the indicated time points. Mock-treated cells were used as controls. Infected and mock-treated cells were fixed and immunostained for anti-flavivirus group antigen, 4G2. The representative images show individual channels of fluorescence microscopy in red (ZIKV-4G2), Blue (DAPI-stained nucleus), and Merge (overlap) images, scale bar, 50 µm. **(B)** Bright-field/ phase contrast micrographs were captured to observe ZIKV-induced cytopathy. Scale bar, 100 µm.


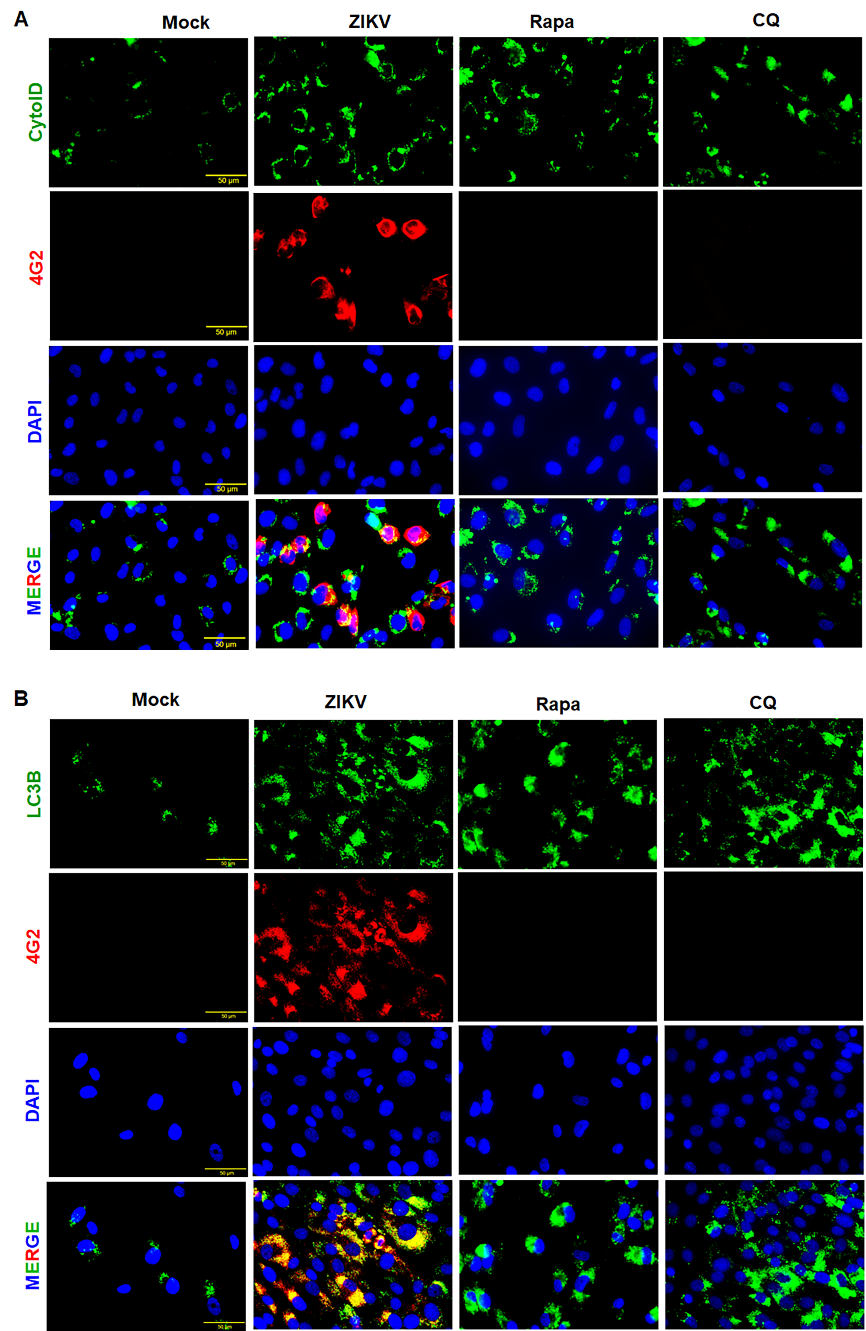


**Supplementary Figure 2:** **ZIKV activates autophagic activity in HTMCs.** HTMCs were infected with ZIKV at an MOI of 1 for 48h. Mock-treated cells were used as controls. **(A)** Autophagic activation was evaluated in ZIKV-infected/mock-treated HTMC by staining with a CYTO-ID^®^ autophagy detection probe, which is specific for autophagosomes. An autophagy activator, rapamycin (Rapa), and an autophagy inhibitor, chloroquine (CQ), without ZIKV infection, were used as autophagy inducer and inhibitor controls, respectively, in these experiments. The representative images show individual channels of fluorescence microscopy in green (CytoID probe^+^ autophagosomes), red (ZIKV-4G2), Blue (DAPI-stained nucleus), and Merge (overlap) images. **(B)** In another set of experiments, ZIKV-infected and mock-treated cells were immunostained with autophagy marker LC3B along with anti-flavivirus group antigen 4G2. Rapa and CQ, without ZIKV infection, were used as autophagy inducer and inhibitor controls, respectively. The representative images show individual channels of fluorescence microscopy in green (LC3B^+^ autophagosomes), red (ZIKV-4G2), Blue (DAPI-stained nucleus), and Merge (overlap) images, scale bar, 50µm.


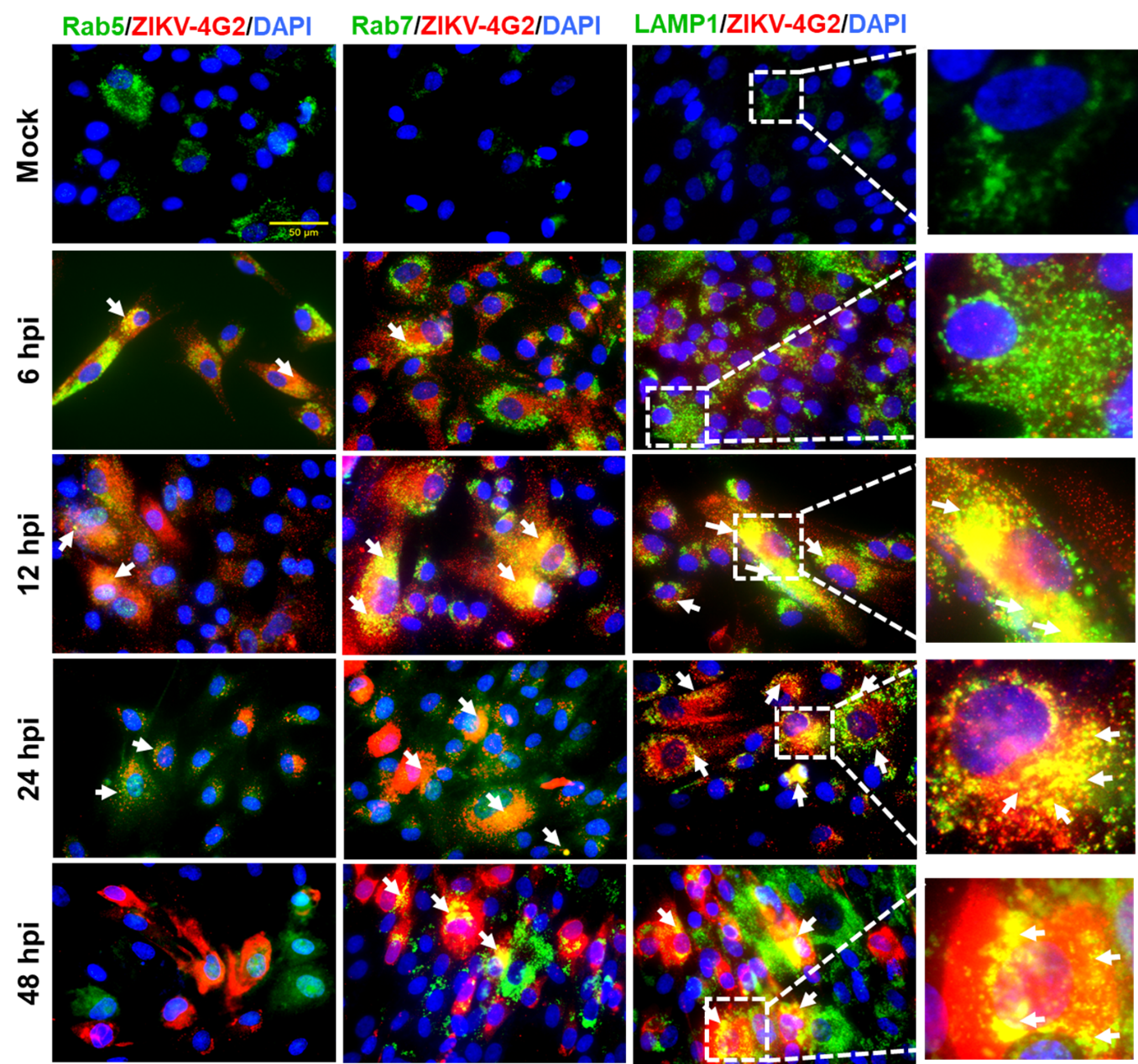


**Supplementary Figure 3: ZIKV blocks autophagic maturation and replicates in late-endosomes/ lysosomes.** HTMCs were infected with ZIKV strain PRVABC59 at an MOI of 1 for the indicated time points. The cells were fixed and coimmunostained for anti-flaviviral E antigen (4G2) antibody (Red) with cellular organelle markers Rab5 (early endosome), Rab7 (late endosome), and LAMP-1 (lysosome) (Green), and DAPI nuclear stain (Blue). A few representative co-localizations of ZIKV (4G2^+^) with organelles at various time points are indicated with white arrows. The insert panels on the right show higher magnification images indicating colocalization of ZIKV with lysosomes (ZIKV^+^LAMP1^+^), scale bar, 50µm.


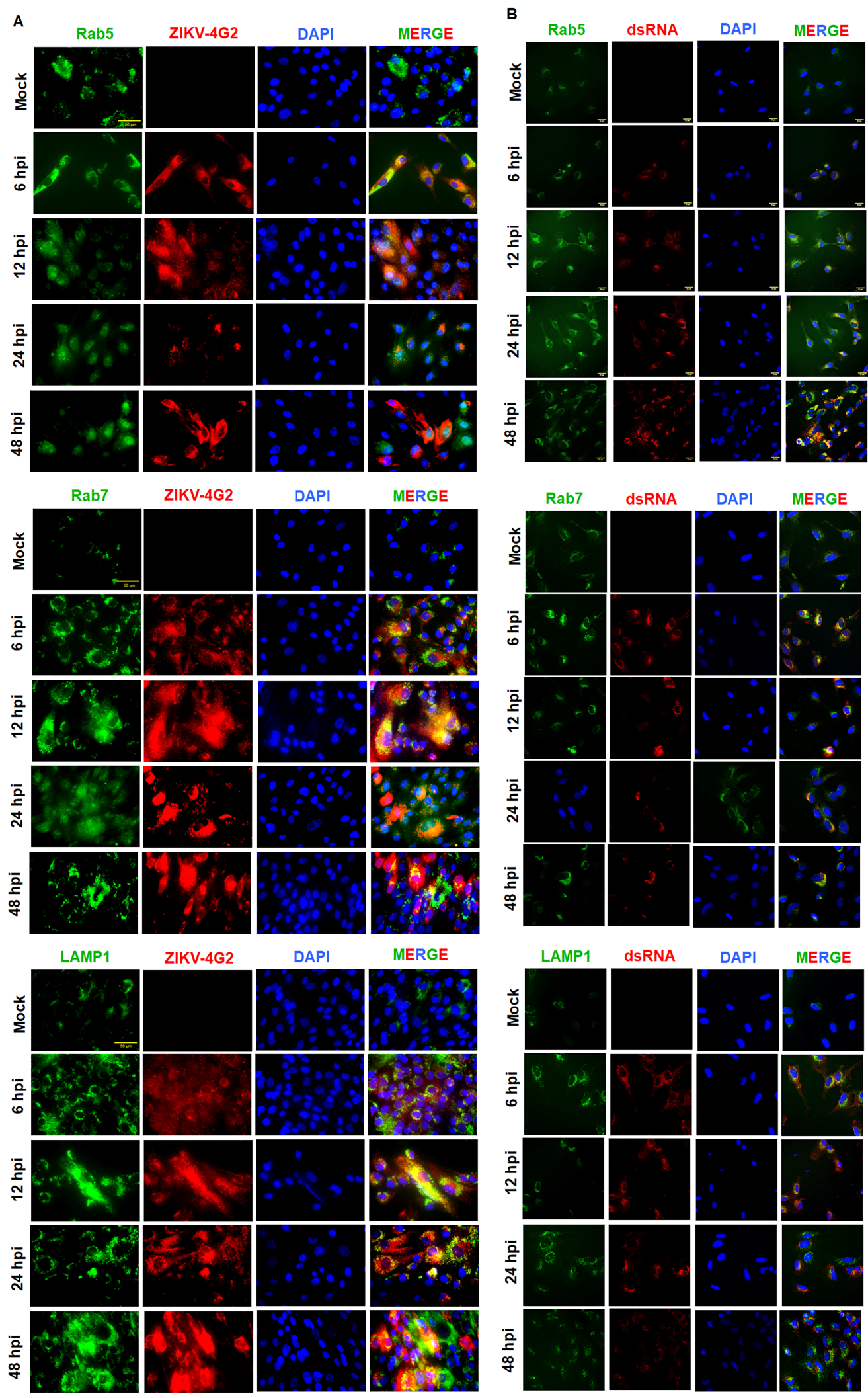


**Supplementary Figure S4: ZIKV replicates in late-endosomes/ lysosomes.** HTMCs were infected with ZIKV strain PRVABC59 at an MOI of 1 for the indicated time points. The cells were fixed and immunostained for **(A)** anti-flaviviral E antigen antibody, 4G2, or **(B)** viral anti-dsRNA antibody along with cellular organelle markers Rab5 (early endosome), Rab7 (late endosome), and LAMP-1 (lysosome). The representative images show individual channels of fluorescence microscopy in green (Rab 5/ Rab 7/ LAMP 1), red (ZIKV-4G2 or dsRNA), Blue (DAPI-stained nucleus), and Merge (colocalization of subcellular organelles with 4G2 or dsRNA) images, scale bar: 50µm (panel A), 25µm (panel B).
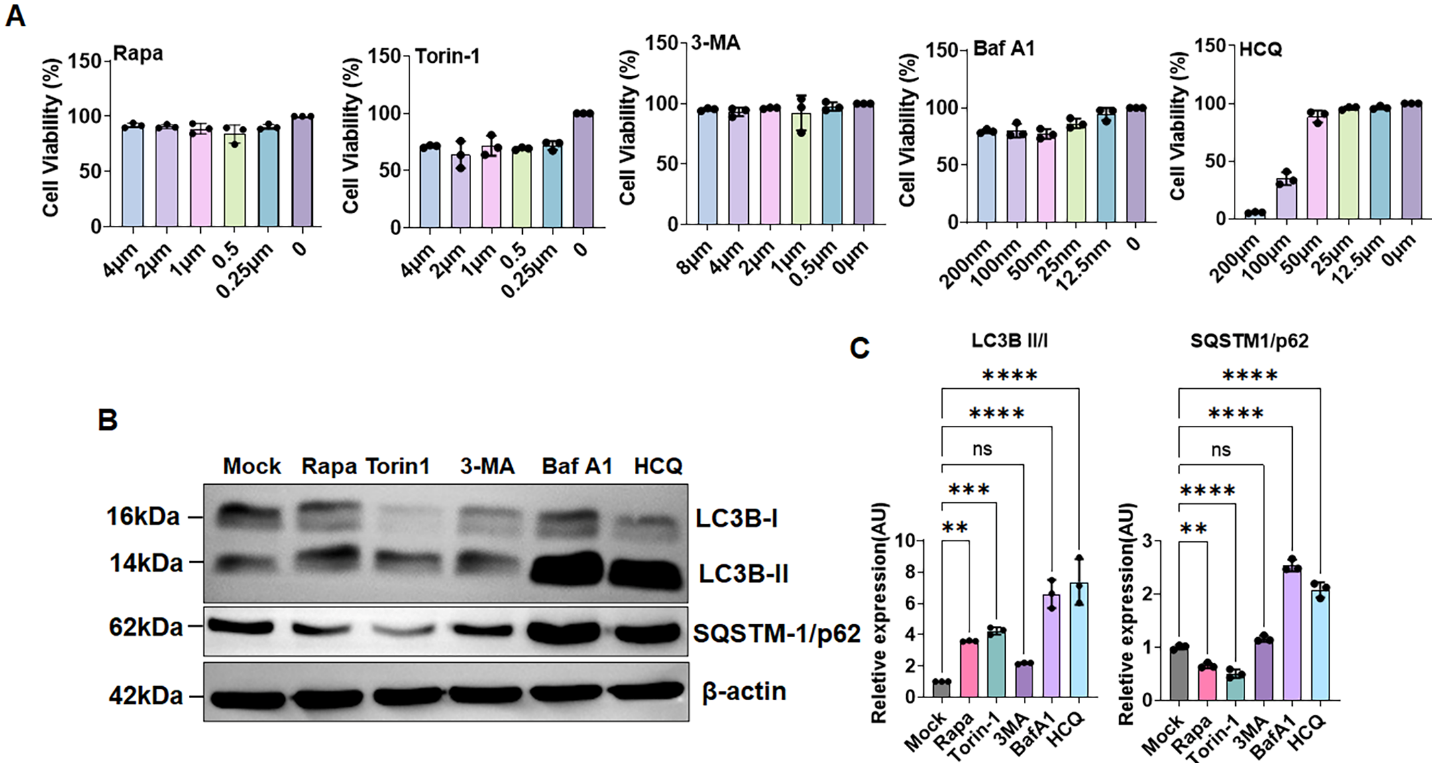


**Supplementary Figure 5: Cytotoxicity assay and autophagy modulation by rapamycin, torin-1, 3-MA, Baf-A1, and HCQ. (A)** HTMCs were treated with indicated doses of autophagy modulators (Rapa, torin-1, 3-MA, Baf-A1, and HCQ), and cytotoxicity assay was performed using the cell proliferation reagent WST-1. Untreated cells (0 µM) were used as controls. **(B)** HTMC were treated with [Rapa (1 µM), Torin 1 (1 µM)] and inhibitors [3-MA (2 μM), Baf-A1 (50 nM), and HCQ (50 μM)] alone without ZIKV infection, and cell lysates were subjected to western blot analysis for LC3B and SQSTM1/p62 proteins. **(C)** Densitometric analysis for ZIKV (Z) NS3, LC3B-II/I, and SQSTM1/p62 proteins (relative to β-actin) (n=3) were performed using ImageJ (mean±SD, ** P < 0.005; *** P < 0.0005; ****, P < 0.0001; One-way ANOVA.


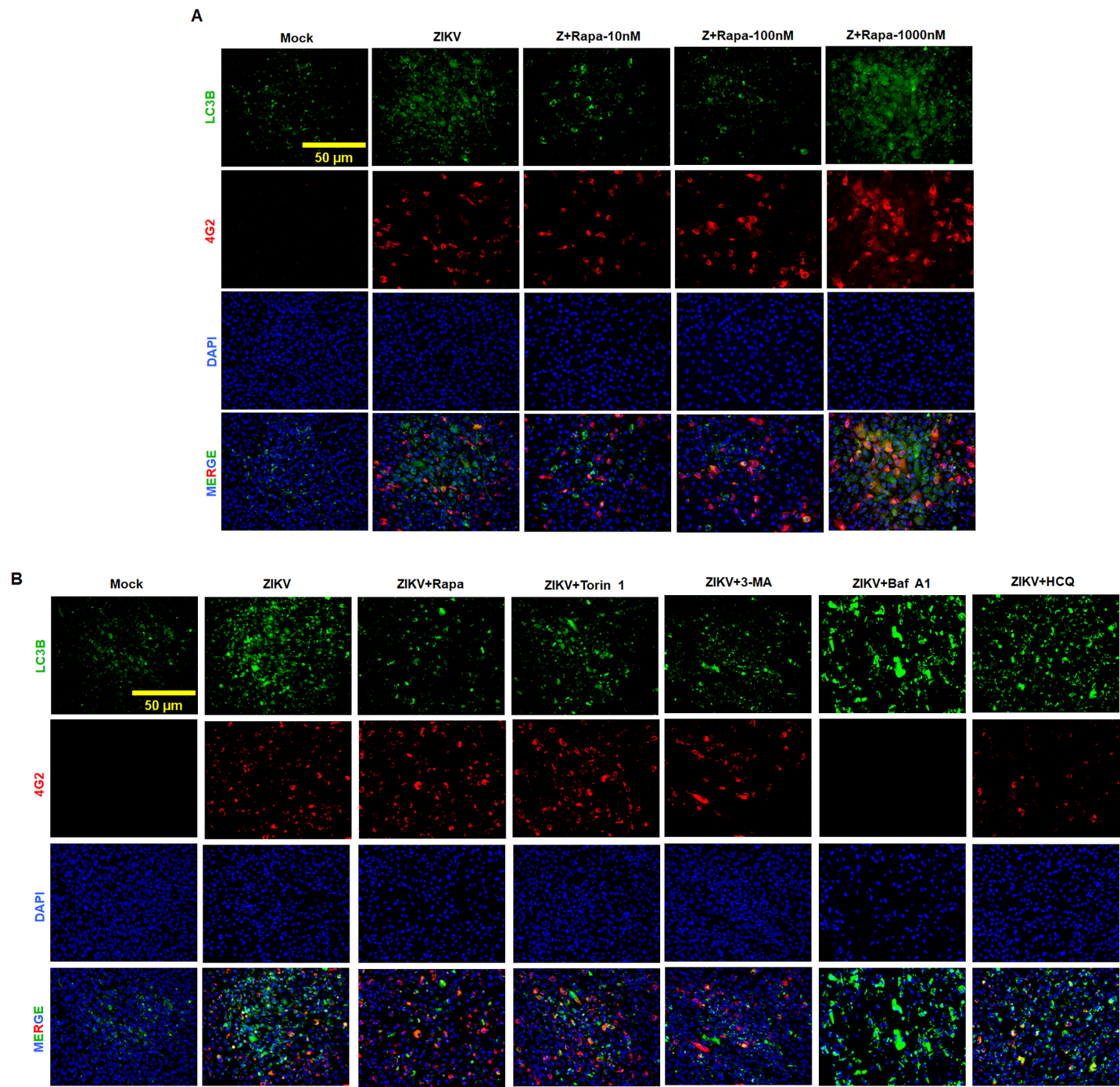


**Supplementary Figure 6: Autophagy induction promotes ZIKV replication, while autophagy suppression restricts viral replication in HTMCs.** **(A)** HTMCs were pretreated with different doses (10, 100, 1000 nM) of autophagy inducer rapamycin (Rapa) followed by ZIKV (Z) infection (MOI:1) for 48h. Mock-treated cells were used as controls. Cells were fixed and immunostained for anti-flavivirus group antigen 4G2 and autophagy marker LC3B. **(B)** In another set of experiments, HTMCs were pretreated with various autophagy activators [Rapa (1 µM), Torin 1 (1µM)] and inhibitors [3-MA (2 μM), Baf-A1 (50 nM), and HCQ (50 μM)] followed by ZIKV infection (MOI: 1) for 48h. The cells were fixed and immunostained for ZIKV E antigen 4G2 and autophagy marker- LC3B. The representative images show individual channels of fluorescence microscopy in green (LC3B), red (ZIKV-4G2), Blue (DAPI-stained nucleus), and merge (overlap) images, scale bar, 50 µm.


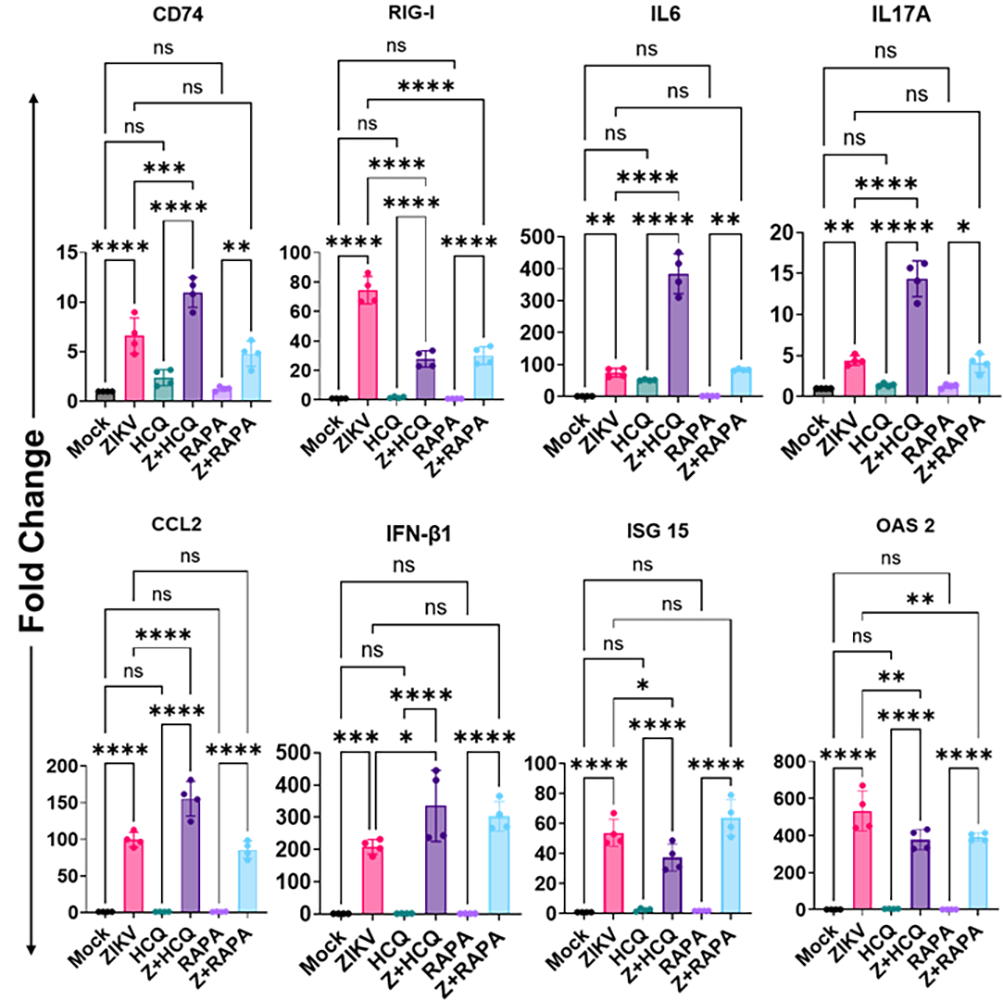


**Supplementary Figure 7: Modulation of HTMC transcripts by HCQ and Rapamycin with or without ZIKV.** HTMC (n=4) was pretreated with either HCQ (50 µM) or Rapamycin (RAPA) (1 µM), followed by ZIKV (Z) infection for 48h. HCQ or Rapa alone and uninfected (Mock) cells were used as controls. Total RNA was extracted and subjected to qPCR analysis for the indicated gene expression. The data represent mean ± SD from four biological replicates. * P < 0.05; ** P < 0.05, ** P < 0.05, ****, P < 0.0001; one-way ANOVA.
